# Asian inland wildfires driven by glacial–interglacial climate change

**DOI:** 10.1073/pnas.1822035117

**Published:** 2020-02-24

**Authors:** Yongming Han, Zhisheng An, Jennifer R. Marlon, Raymond S. Bradley, Changlin Zhan, Richard Arimoto, Youbin Sun, Weijian Zhou, Feng Wu, Qiyuan Wang, George S. Burr, Junji Cao

**Affiliations:** ^a^State Key Laboratory of Loess and Quaternary Geology, Center for Excellence in Quaternary Science and Global Change, Chinese Academy of Sciences, Xi’an 710061, China;; ^b^Key Laboratory of Aerosol Chemistry and Physics, Institute of Earth Environment, Chinese Academy of Sciences, Xi’an 710061, China;; ^c^School of Human Settlements and Civil Engineering, Xi’an Jiaotong University, Xi’an 710049, China;; ^d^Interdisciplinary Research Center of Earth Science Frontier, Beijing Normal University, Beijing 100875, China;; ^e^Open Studio for Oceanic-Continental Climate and Environment Changes, Qingdao National Laboratory for Marine Science and Technology, Qingdao 266061, China;; ^f^School of Forestry & Environmental Studies, Yale University, New Haven, CT 06511;; ^g^Climate System Research Center, Department of Geosciences, University of Massachusetts, Amherst, MA 01003;; ^h^School of Environmental Science and Engineering, Hubei Polytechnic University, Huangshi 435003, China

**Keywords:** biomass burning, Quaternary climate, carbon cycle, high-intensity fires, soluble iron

## Abstract

Wildfire can influence climate directly and indirectly, but little is known about the relationships between wildfire and climate during the Quaternary, especially how wildfire patterns varied over glacial–interglacial cycles. Here, we present a high-resolution soot record from the Chinese Loess Plateau; this is a record of large-scale, high-intensity fires over the past 2.6 My. We observed a unique and distinct glacial–interglacial cyclicity of soot over the entire Quaternary Period synchronous with marine δ^18^O and dust records, which suggests that ice-volume-modulated aridity controlled wildfire occurrences, soot production, and dust fluxes in central Asia. The high-intensity fires were also found to be anticorrelated with global atmospheric CO_2_ records over the past eight glacial–interglacial cycles, implying a possible connection between the fires, dust, and climate mediated through the iron cycle. The significance of this hypothetical connection remains to be determined, but the relationships revealed in this study hint at the potential importance of wildfire for the global climate system.

## Significance

We reconstructed a unique record of soot variations from a classic Chinese loess section that reflects regional-to-continental scale high-intensity fires in central Asia over the entire Quaternary. This study shows cyclicity of wildfire over glacial–interglacial intervals. High-intensity wildfires were more common and dust loads were high during dry and cold glacial periods, implying a synchronous response to climate change. Our study suggests potential linkages among wildfire, mineral dust, marine biogeochemical cycles, atmospheric CO_2_, and glacial–interglacial climate change. Understanding these connections among earth systems provides insights into climate dynamics during the geological past and may improve predictions for the future.

Wildfire has been an intrinsic feature of Earth systems for hundreds of My (1), and fires can enhance or mitigate climate change directly and indirectly (2). For example, modern wildfire observations have highlighted influences on atmospheric chemistry caused by the emissions of greenhouse gases—carbon dioxide (CO_2_), methane (CH_4_), carbon monoxide (CO), and aerosols—organic carbon (OC) and black carbon (BC) (3, 4). Other effects of wildfire include changes in land surface albedo, clouds, and precipitation (5). Paleoclimate studies have shown that wildfire has affected global biome distributions, reshaped the human world (6), and influenced Miocene C4 vegetation (7). Increased light absorption caused by BC in snow and snowmelt may have led to glacial retreat at the end of the Little Ice Age (8). In the past decade an additional effect of wildfire on climate has been recognized; that is, the fires supply soluble, bioavailable iron to the oceans, in addition to other micronutrients such as organic N and P (9, 10), possibly promoting the growth of marine phytoplankton and affecting the concentration of atmospheric CO_2_ (11, 12). However, this effect has never been considered in the context of paleowildfire.

Understanding what controls wildfires (e.g., global warming, fire suppression, and aridification), especially climate–wildfire interactions, will improve models and our ability to understand and predict changes in the earth–climate system. Important insights can be obtained from long-term, high-time-resolution, synchronized records of fire activity and climate across a broad range of climate states, such as those climate extremes experienced during the glacial–interglacial Quaternary Period.

Previous efforts to investigate wildfire–climate interactions have made use of charcoal and BC for paleowildfire reconstructions, and those records were interpreted in relation to temporal changes in temperature and precipitation (13, 14). However, charcoal and BC proxies for wildfire reconstruction do not have clear connections to climate. For example, both high and low temperatures, and dry and wet climatic conditions, have been linked to wildfire occurrences (14, 15). This may be because charcoal and BC only represent local burning conditions (16). Although efforts have been made to produce composite records of continental and global fire histories (13, 14), they have been limited to relatively short-term records, such as those since the Last Glacial Maximum. Long-term wildfire records covering glacial–interglacial cycles are still notably sparse, and the orbital scale pattern of wildfire behavior is still unknown.

Recently, methods have been developed to measure char and soot, which are two subtypes of BC that have more clear-cut connections to climate (17). Modern wildfire observations show that the proportions of char and soot vary with climatic conditions due to the effects of climate on combustion efficiency (18) (*SI Appendix*, section 3). Soot particles, produced via a high-temperature gas-to-particle conversion under dry conditions (18), are predominantly in the submicrometer mode and therefore reflect regional to continental high-intensity (flaming) fires (17). In contrast, char particles are larger combustion residues (>1 µm) similar to charcoal, that reflect local low-intensity smoldering fires (17). The ability to distinguish between regional high-intensity fires and more local fires provides us with an approach for investigating wildfire–climate interactions.

**Fig. 1.**
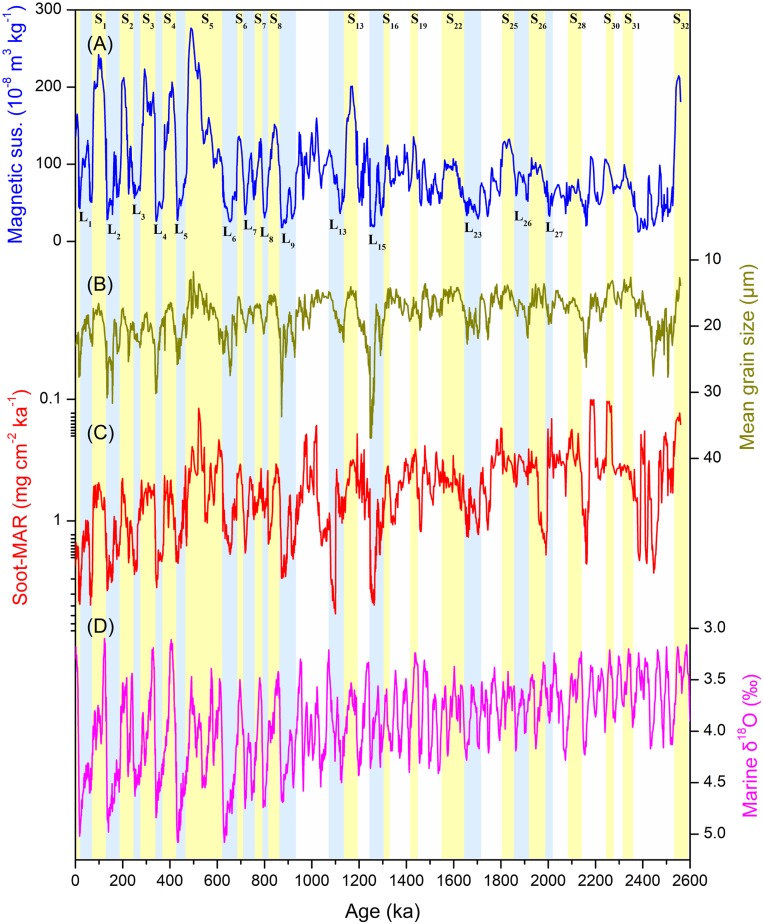
Quaternary high-intensity fires indicated by soot-MARs reconstructed from the Luochuan section of the CLP compared with other climatic parameters. (*A*) MS from Luochuan, an indicator of precipitation on the CLP (19); (*B*) MGS from Luochuan, an indicator of wind strength and the aridity of the dust source area (26); (*C*) Soot-MAR from Luochuan, indicating high-intensity fires; (*D*) marine benthic oxygen stable isotopes, an indicator of ice volume (22). The yellow shaded areas indicate paleosol (S) layers, corresponding to interglacial periods, while the light blue shaded areas indicate loess (L) layers, corresponding to glacial periods. Below L9, not all layers reflecting glacial–interglacial cycles are shown with different colors; this was done to highlight only the main patterns in the cycles. Note: the scales for the MGS, soot-MAR, and marine δ^18^O are reversed.

Windblown sediments on the Chinese Loess Plateau (CLP) consist of alternating layers of loess and paleosol that span the entire Quaternary (19, 20) (*SI Appendix*, section 1 and Fig. S1). Glacial–interglacial cycles are reflected in magnetic susceptibility (MS, Fig. 1*A*) and grain-size variations (Fig. 1*B*) of loess–paleosol sequences, which have been used to reconstruct East Asian monsoon (EAM) changes between warm, humid interglacials, and cold, dry glacials (20). Extensive studies of Chinese loess sections have demonstrated teleconnections between the EAM and large-scale atmospheric and oceanic processes (20).

In this study, we measured BC and its two subtypes, soot and char, in sediments (21) from the classical Luochuan section (35.8° N, 109.4° E, *SI Appendix*, Fig. S1) of the central CLP. The first objective of the study was to reconstruct the history of Quaternary wildfires and to differentiate continental high-intensity fires from more local, low-intensity smoldering combustion. The chronology of this section was generated by matching mean grain-size variations with the deep-sea δ^18^O stack (22) and previous representations of stacked quartz grain-size records (23) (*Materials and Methods* and *SI Appendix*, Fig. S2). We present a soot-based record of high-intensity fires that demonstrates clear glacial–interglacial variations during the Quaternary. We also observed a consistent anticorrelation between high-intensity fires and atmospheric CO_2_. We propose that the wildfires contributed soluble iron and other substances to the global ocean by direct emission (11, 12) and via atmospheric processes (12, 24, 25), raising the possibility of some involvement in glacial–interglacial climate change.

## Results and Discussion

### Glacial–Interglacial Cyclicity of High-Intensity Fires Controlled by Ice-Volume-Modulated Aridification.

Throughout the Quaternary, high-intensity fires––as indicated by soot mass accumulation rates (MARs)––show a clear and consistent relationship to climate. Elevated soot-MARs occur during dry glacial periods and low values occur during wet interglacials (Fig. 1*C*). Soot concentrations normalized with lithogenic titanium (soot/Ti ratio, *SI Appendix*, Fig. S3) to remove the influence of terrestrial input (*Materials and Methods*) covary with mean grain size of dust particles (Fig. 1*B*), an indicator of wind strength and the aridity of the loess source area (26). Stronger winds and/or increased high-intensity fires during glacial times would result in high proportions of soot particles, which are easily transported through the atmosphere (17). An abrupt increase in both soot-MARs and Ti-normalized soot concentrations was observed after ∼500 ka (*SI Appendix*, Fig. S3), coincident with the rapid aridification of central and interior Asia (27, 28); this indicates that the CLP records reflect the regional to continental scale transport of soot.

Local low-intensity fires, as indicated by char-MARs, followed the temporal pattern exhibited by the dust-MARs, which are an indication of aridification of the loess source area (26), and by the soot-MARs at Luochuan during the glacial–interglacial cycles prior to the Mid-Pleistocene climate transition (before ∼900 ka) (*SI Appendix*, Fig. S4). The char cycles are not as distinct as those for soot, however, and after 900 ka, the char-MARs did not follow consistent glacial/interglacial trends. For example, the char-MAR values for the S0, S1, and S5 paleosol layers were higher compared with those in nearby loess layers (*SI Appendix*, Fig. S4). This result is consistent with a more localized production of char because the smoldering fires that mainly produce char can occur in relatively wet conditions. In addition, the different formation pathways of char and soot (17, 18) may also influence the distributions of these substances. That is, soot is mainly produced by high combustion efficiency fires under dry conditions while char, on the other hand, is mainly produced during low-efficiency fires in wetter conditions but also can be emitted along with soot in different stages of a single high-intensity flaming fire. (*SI Appendix*, section 3). Thus, the temporal variations in soot have much clearer climatic implications than those of char.

Char/soot ratios (*SI Appendix*, Fig. S4*D*) exhibit clear glacial–interglacial cycles, which positively covary with MS (*SI Appendix*, Fig. S4*E*), an indicator of the East Asian summer monsoon (19). This is in good agreement with the pathways by which char and soot are produced, as mentioned above (17, 18) because stronger summer monsoons correspond with wetter conditions, favoring char production. This explanation is also supported by principal component analysis (*SI Appendix*, Table S1), which shows that the division between smoldering and high-intensity fires, indicated by char- and soot-MARs, respectively, is related to the available moisture. Correspondence between soot-MARs and dust-MARs (*SI Appendix*, Table S1) strongly supports the possibility that high-intensity wildfires occurred more frequently when the climate was dry and combustion efficiencies were high (17, 18). Indeed, the combustion efficiency of modern fires has been observed to be anticorrelated with monthly precipitation and is unrelated to ambient monthly air temperature (3).

Over the duration of the full Quaternary CLP record, we observed an increase in wildfires (Fig. 1*C* and *SI Appendix*, Fig. S4). This long-term pattern is in good agreement with a drying trend in the dust source regions (27) and in the CLP (29), which was associated with the expansion of the Northern Hemisphere ice sheets (22) (Fig. 1*D*). Continuous increases in wildfires during the Pleistocene also have been found in previous studies of both terrestrial and marine sediments with low time resolution (30, 31).

Although several studies have reported wildfire histories on the CLP (30), our record shows clear glacial/interglacial cycles. This is because previous studies did not distinguish between the occurrences of regional-high intensity versus local-smoldering fires that can be inferred from the BC components of soot and char. Our soot-MAR high-intensity fire record contrasts with global charcoal-based wildfire records (14, 32), from which it has been concluded that there were more wildfires during wet and warm periods over the past 70,000 y (14). Our soot-MAR record is analogous to BC or microcharcoal records in marine sediments from the coast of southern Africa, and at other sites (15, 33), but those records have lower temporal resolution and a shorter duration than our record. The marine records also represent sites distant from biomass burning sources. Some studies of marine records found precession-driven increases in biomass burning during glacial periods (15). For the CLP, we propose that the observed glacial–interglacial biomass burning cycles were mainly driven by changes in aridity.

**Fig. 2.**
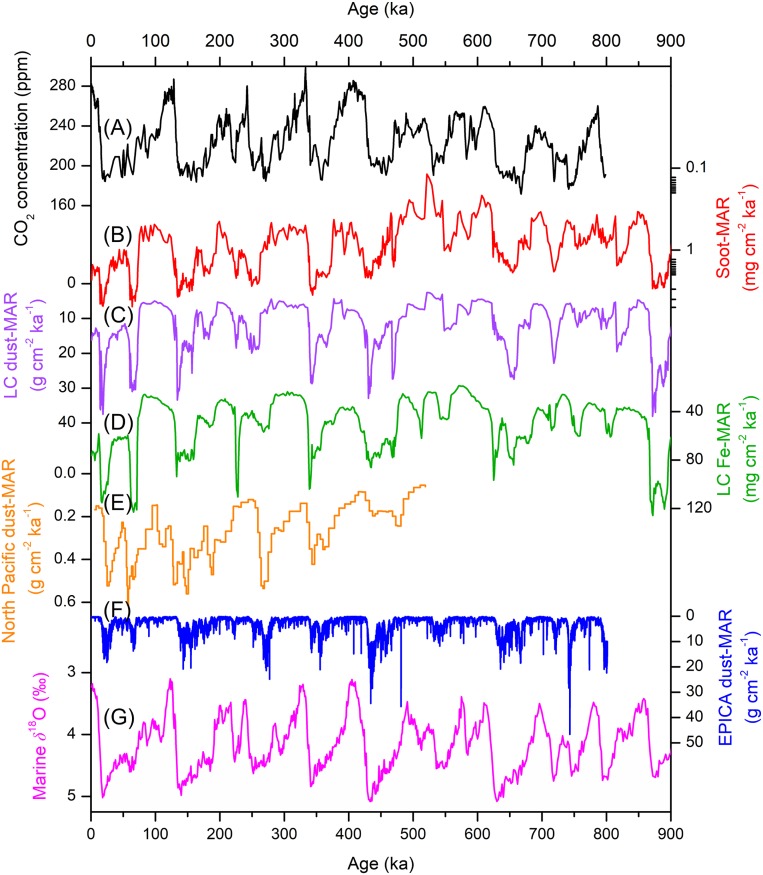
Comparison of soot-MARs with atmospheric carbon dioxide (CO_2_) concentrations and regional to global dust- and iron-MARs over the past eight glacial–interglacial cycles. (*A*) Atmospheric CO_2_ concentrations (37); (*B*) Soot-MARs reflecting regional high-intensity fires; (*C* and *D*) Dust- and iron-MARs for Luochuan (LC); (*E*) North Pacific dust-MAR record (34); (*F*) EPICA Dome C ice-core dust-MARs (35); (*G*) Marine benthic oxygen stable isotopes (22).

Temporal patterns in our CLP soot-MAR record are strongly correlated with global ice volume as reflected in the marine δ^18^O record (22) and dust flux (34, 35) (Fig. 2 *B*–*G*). These coherent trends imply that high-intensity fires and soot production are both connected with climate. The synchrony of our soot record with the marine δ^18^O record suggests that ice-volume-modulated aridity exerted control over fire occurrences in central Asia during the Quaternary. Increases in global ice volume have been associated with stronger westerly winds, a strengthening of the East Asian winter monsoon (36), and increased aridity in both the East Asian dust source areas and the CLP (26). These conditions all favor high-intensity combustion (18) and lead to the production and transport of both soot and dust.

### Wildfire, Climate Change, and Carbon Sequestration.

The continental high-intensity fire record we produced is anticorrelated with atmospheric CO_2_ concentrations reconstructed from ice cores over the past eight glacial–interglacial cycles (37) (Fig. 2 *A* and *B*). The relationships between wildfire, marine δ^18^O, dust, and atmospheric CO_2_ records (Figs. 1 and 2) imply interactions between wildfire and glacial–interglacial climate. The effects of wildfire on greenhouse gas emissions and surface albedo can be offset by several decades due to natural successional cycles of greenhouse gases caused by the regeneration of vegetation (1), and the radiative effects of aerosols from fire emissions would be limited to a few weeks or months at most (38). Therefore, these effects would not be expected to connect wildfires with long-term glacial–interglacial climate change.

Previous studies have raised the possibility that desert dust affected glacial–interglacial climate through the atmospheric deposition of soluble iron to the ocean (39, 40). According to the “iron hypothesis” (41), soluble iron from deposited mineral dust stimulated ocean primary productivity in regions of the ocean where the levels of classical nutrients were high but concentrations of chlorophyll were low, leading to the drawdown of atmospheric CO_2_. Wildfire is suggested to be another main contributor of soluble iron to the modern oceans, possibly rivaling that from mineral dust (11, 12), and the observed anticorrelation found between our long-term wildfire record and atmospheric CO_2_ suggests the possible involvement of fires in the iron cycle. The covariations between high-intensity fires and dust loadings at our site and other sites (Fig. 2 *B*–*F*) indicate that the wildfire and mineral dust contributions of soluble Fe to the oceans were connected in time. Indeed, covariations between dust and BC also have been observed in studies of modern aerosols (12, 42) and ice cores (43).

Spectral frequency analyses (*SI Appendix*, Fig. S6) show significant periodicities for marine δ^18^O (22), atmospheric CO_2_ (37), Luochuan dust-MARs, and Luochuan soot-MARs over the past 800 ka, all exceeding the 90% confidence interval for 23, 41, and 100 ka. The expansion of Northern Hemisphere ice sheets during glacial periods was synchronous with increases in both dust production and soot emissions from high-intensity wildfires (Fig. 2 *C*–*G*). Taken together, these relationships imply that ice-volume-modulated aridity not only affected the production of desert dust, but also the occurrence of high-intensity fires.

We also note that a period of low soot-MARs (∼621–474 ka) over the past 900 ka (*SI Appendix*, Fig. S5) includes a mild glacial event, marine isotope stage (MIS) 14 (568–528 ka) and two adjacent interglacial periods, MIS 15 (621–568 ka) and 13 (528–474 ka). The marine δ^18^O values for MIS 14 were relatively low (22) compared with other glacial times, and together with MIS 15 and 13, the low soot-MARs period corresponds well with the low δ^18^O values that indicate low ice volume (22). After this period there was an increasing trend in soot-MARs (Fig. 2*B* and *SI Appendix*, Fig. S5*B*), and there was drying on the CLP as indicated by the variations of dust-MARs in the region (Fig. 2*C*). This drying trend was also evident in downwind areas such as the North Pacific Ocean (34) (Fig. 2*E*), where the dust record reflects the drought history of the dust source areas in Central Asia (28, 34). These findings are consistent with the idea that ice volume was an important influence on both drought in the CLP and wildfire occurrence.

Marine benthic δ^13^C has been suggested as a possible proxy for pCO_2_ (44). In order to investigate potential relationship between wildfire and pCO_2_ over the past 900 ka, we compared our Luochuan soot-MAR record with marine records of benthic δ^13^C from the North Atlantic (45), Equatorial Pacific, and South Atlantic (46) (*SI Appendix*, Fig. S5). This comparison showed overall that the low soot-MARs around 621–474 ka corresponded with high benthic δ^13^C even though some spikes of relatively low benthic δ^13^C values were evident. It is especially notable that the highest benthic δ^13^C values over the past 900 ka were found during MIS 13 (*SI Appendix*, Fig. S5). Following that, marine benthic δ^13^C values decreased, which suggests a decrease in pCO_2_ concentrations (44), and this trend corresponds with an increase in the Luochuan soot-MARs (*SI Appendix*, Fig. S5). These findings support our suggestion that wildfire should be included in future assessments of the iron hypothesis.

In terms of significance, the fertilization of the oceans by iron was suggested to account for no more than half the observed glacial atmospheric CO_2_ drawdown (47–51). In addition to the stimulating effects of the iron inputs from dust and biomass burning proposed here, the enhanced glacial marine productivity also may have been a response to the inputs of nutrients from upwelling, mixing (40, 52), and volcanic eruptions (53, 54). Indeed, there are various other mechanisms (40, 47, 49, 50) including physical changes in ocean circulation, which have been suggested to be the most important causes for the early stages of glaciation (49). In addition, land carbon storage is another factor that can influence glacial–interglacial variations of atmospheric CO_2_ (47, 49). Nonetheless, covariations in the soot and dust records indicate that dust and fires provided micronutrients to the oceans at roughly the same time. Therefore, the soluble iron from fires can be regarded as a supplement to the mineral dust iron, which at times may have affected marine productivity through the “biological pump.”

Overall, our wildfire record shows clear evidence of glacial–interglacial cyclicity of high-intensity fires during the Quaternary. In addition to causing greater dust emissions, ice-volume-modulated aridification during glacial periods evidently increased the occurrence of high-intensity wildfires. This highlights a response of wildfires to Quaternary glacial/interglacial climate change. Although the mechanisms influencing the glacial atmospheric CO_2_ are complex (40, 49) and not fully understood, our study raises the intriguing possibility that wildfire contributed to Quaternary climate change through its involvement in marine biogeochemical cycles. Our 2.6-My record of soot and char from the CLP provides insights into fires in relation to environmental change in central Asia, and the results may serve as a basis for further investigations into the linkages between wildfire and climate change.

## Materials and Methods

A total of 1,339 sediment samples were collected at 10-cm intervals from the Luochuan section in August 2009. The samples were kept in a cold storage facility at the Institute of Earth Environment, Chinese Academy of Sciences (IEECAS) until they were analyzed.

The low-field MS of each sample was determined with a Bartington MS2 susceptibility meter (470/4,700 Hz, Bartington Instruments). Results were given in units of 10^−8^⋅m^3^⋅kg^−1^. In preparation for grain-size analyses, bulk samples were pretreated with 30% hydrogen peroxide (H_2_O_2_) to remove organic matter and with 10% hydrochloric acid to remove carbonates and iron oxides. The grain sizes of these pretreated samples were then measured with the use of a Malvern 2000 Laser Instrument (Malvern Instruments, Ltd.). The bulk density of the sediments was measured by using an oil-soaked method (55).

An initial chronology was generated using pedostratigraphic and paleomagnetic correlation. Several age-control points were taken at the S_0_/L_1_ boundary at 11 ka B.P., S_1_/L_2_ boundary at 128 ka, Brunhes/Matuyama at 0.78 Ma, top of the Jaramillo at 0.99 Ma, bottom of the Jaramillo at 1.07 Ma, top of the Olduvai at 1.77 Ma, bottom of the Olduvai at 1.95 Ma, and top of the Gauss at 2.58 Ma (56). Grain-size variations in the Chinese loess-paleosol sequences can be well correlated with marine oxygen-isotope records due to a strong coupling between global ice volume and winter monsoon intensity (23). From this correlation we generated a refined chronology by matching mean grain-size variations with a deep-sea δ^18^O stack (22) and the orbitally tuned loess grain-size time series (23, 57). Good cycle-by-cycle correlations of LC (Luochuan) grain-size variations with the stacked benthic δ^18^O and previous representations of the stacked quartz grain size (23, 57) suggest that our age model provides robust constraints on glacial–interglacial changes over the past 2,600 ka (*SI Appendix*, Fig. S2).

BC, char, and soot concentrations were determined using the IMPROVE (Interagency Monitoring of Protected Visual Environments) protocol after chemical pretreatment (17, 21, 58). Please refer to *SI Appendix*, section 2 and Han et al. (58) for details. Mass accumulation rates (MARs, g cm^−2^⋅ka^−1^) of dust, BC, char, and soot were calculated using the following equations:

dust-MAR=BD×LDR, [1]

LDR=H/T, [2]

MAR(BC,char,or soot)=C×dust-MAR, [3]

where BD (g cm^−3^) is the bulk density of each loess sample, LDR (cm ka^−1^) is the dust deposition rate, H (cm) is the thickness of the sampled layer, T (ka) is the duration of its deposition, and C (mg g^−1^) is the concentration of BC, char, or soot.

Uncertainty evaluations were conducted using an error propagation method. Three uncertainties were considered: 1) quantification of BC, char, and soot; 2) BD measurements; and 3) dust deposition rate. The error estimations for BC, char, and soot measurement were based on the minimum detection limit of the DRI Model 2001 carbon analyzer and the replicate analyses which were performed at a rate of 1 per group of 10 samples (see *SI Appendix*, section 2 for details). The error for the dust deposition rate was estimated based on a regression between the LDR and particle size of loess. We assumed that the LDR was mainly determined by the wind strength because that largely determines the particle size of loess; that is, stronger winds carry a greater amount of larger particles, and therefore the dust deposition rate is higher. The uncertainty of the LDR was estimated as the coefficient of variance of the root-mean-square residual (CV-RMSR) in the regression between DDR and particle size. The resulting linear calibration (LDR = particle size × 0.7826–8.355, *r*^2^ = 0.585) has a CV-RMSR of 0.37. The calculated uncertainties for BC, char, and soot-MARs were 37.5–37.6%, 37.9–61.0%, and 37.9–38.0%, respectively.

To determine whether soot concentrations in sediments were affected by inputs of terrigenous material, the data were normalized by dividing the soot concentrations by those of lithogenic elements (15). The concentrations of typical lithogenic elements, including Al, Ti, Zr, Si, and Fe, were measured with a Philips PW4400 X-ray Fluorescence spectrometer at the IEECAS. For this study, only Ti was selected for the normalization procedure (*SI Appendix*, Fig. S3).

### Data Availability.

All data for the Luochuan section used in this study, including MARs for soot, char, and dust; MS; and mean grain size (MGS) are provided in *SI Appendix*, Table S2.

## Supplementary Material

Supplementary File
