# Supplementary material for: Asian inland wildfires driven by glacial–interglacial climate change
Source: Proc Natl Acad Sci U S A. 2020 Feb 24;117(10):5184–9. doi: 10.1073/pnas.1822035117 (PMC7071868; doi:10.1073/pnas.1822035117)
Supplement: Supplementary File [file pnas.1822035117.sapp.pdf]

PNAS
www.pnas.org

Supplementary Information for

Asian inland wildfires driven by glacial-interglacial climate change

Yongming Han, Zhisheng An*, Jennifer R. Marlon, Raymond S. Bradley, Changlin Zhan, Richard Arimoto, Youbin Sun, Weijian Zhou, Feng Wu, Qiyuan Wang, George S. Burr, Junji Cao*

*Corresponding authors: Zhisheng An and Junji Cao

Email: anzs@loess.llqg.ac.cn or cao@loess.llqg.ac.cn

This PDF file includes:

Materials and Methods (supplemental)

Figs. S1 to S6;

Tables S1 to S2

SI References

www.pnas.org/cgi/doi/10.1073/pnas.1822035117

Materials and Methods (supplemental)

1. Background and sampling location

The Chinese Loess Plateau (CLP) contains continuous loess-paleosol sequences spanning the entire Quaternary period (1, 2). Loess deposits on the CLP are predominantly eolian dust transported from the Asian Gobi/sandy deserts (3-5) by westerly winds associated with the winter monsoon (2, 6). The climate of the CLP is largely controlled by the alternating influences of the summer and winter monsoons. Cold-dry conditions in winter are driven by continental high-pressure systems while warm-humid summers are associated with moisture flow from low-latitude oceans (7, 8).

Chinese loess sequences record alternating intensities of the East Asian summer and winter monsoons. When the winter monsoon dominates, yellow-brown loess is deposited. When the summer monsoon dominates, a significant drop in the sedimentation rate occurs, with a decrease in grain size and an increase in weathering, producing red-brown paleosols. Alternating loess and paleosol layers represent a succession of glacial (cold and dry) and interglacial (warm and wet) environments. CLP loess profiles preserve long-term, continuous records of East Asian monsoon (EAM) variations, and reconstructions based on these records provide important insights into past global climate change (7, 9-11).

The modern CLP climate is semi-arid to sub-humid and is controlled by monsoonal atmospheric flow. This is expressed as a northwestward decrease in annual precipitation and temperature over the CLP. The Luochuan loess-paleosol section (35.8°N, 109.4°E; ~133 m in thickness) is located on the central CLP and is a classic and well-studied type deposit (1, 12). In the Luochuan region, the mean annual temperature is ~10°C and the mean annual precipitation is ~600 mm.

2. BC, char and soot analysis and quality assessment and control

The IMPROVE (Interagency Monitoring of Protected Visual Environments) protocol (13) was used for black carbon (BC, also sometimes termed elemental

carbon, EC), char, and soot determinations after chemical pretreatment of the samples (14-16). In brief, the sediment samples were dried in an oven at 40 °C for 2 days and then ground using a mortar and pestle so that the particles passed through a 63 μm sieve. About 500 ± 50 mg of each sample was weighed and sequentially pretreated with 2 M HCl, 6 M HCl and 48% HF (1:2, v/v), and 4 M HCl to remove carbonates, minerals, and metal oxides. The residues were then filtered onto pre-fired (850°C, 3 hours) 47-mm quartz filters (Whatman™, Maidstone, UK).

A DRI Model 2001 Thermal/Optical Carbon Analyzer was used for carbon analysis. A 0.526 cm^2 circular punch from the filter was placed into an oven and heated in steps to produce eight carbon fractions as follows: four organic carbon fractions (OC, comprising OC1, OC2, OC3 and OC4, which evolved at 140, 280, 480 and 580 °C in a pure helium inert atmosphere), one pyrolyzed organic carbon fraction (POC, produced during heating in the inert atmosphere and monitored by measuring the return of the laser reflectance to its initial value) and three EC fractions (EC1, EC2, and EC3, which evolved at 580, 740, and 840 °C in 2% oxygen and 98% helium atmosphere, respectively). The evolved carbon gases were oxidized to CO_2 and then reduced to CH_4 for detection with a flame ionization detector (FID). The IMPROVE protocol defines BC (or EC) as the sum of the EC fractions minus POC (17). Han et al. (18) defined char as EC1 minus POC and soot as the sum of EC2 and EC3.
The DRI Model 2001Thermal/Optical Carbon Analyzer (AtmAA Inc., CA, USA) was calibrated daily with known quantities of methane. Standard reference samples (aerosols) were analyzed for calibration purposes each day before the carbon analyzer was operated. Replicate analyses were performed at a rate of one per group of 10 samples. The difference in comparison with the average values for all of these replicate analyses (CV, coefficient of variation) was < 10% for EC and < 12% for char and soot.

The uncertainty (Unc_i) for BC, char, and soot concentrations was calculated using the formula below:

$$\mathrm{Unc}_i = \sqrt{\left(CV \times c_i\right)^2 + MDL^2} \qquad \text{(Eq. S1)}$$

where c_i = concentration of initial analysis; *MDL* = minimum detection limit; CV = coefficient of variance, from which the upper limits of 10% for BC and of 12% for char and soot from the replicate analyses were taken as representative for all samples in this study.

The MDLs of the DRI Model 2001 carbon analyzer for OC and EC (0.45 and 0.06 μg cm^{-2}, respectively) are given in the standard operating procedure for the IMPROVE_A method (19); these were based on the analyses of 214 blank quartz-fiber filters. The MDLs for char and soot were 0.06 and 0.03 μg cm^{-2}, respectively, and these values were calculated from the analyses of 20 blank quartz-fiber filters at the Institute of Earth Environment, Chinese Academy of Sciences (IEECAS). The MDLs (μg cm^{-2}) were then converted to a mass basis (mg g^{-1}) using a nominal sample weight.

3. Different proportional emissions of char and soot from high intensity wildfires and smoldering combustion

High temperature (flaming) and smoldering combustion are typically regarded as the two main fire types, and they can be distinguished based on their combustion efficiency (CE) or modified combustion efficiency (MCE) (20, 21). The latter is more easily measured than CE, and it is defined as the ratio of measured emitted CO_2 to the sum of CO and CO_2:

$$\triangle CO_2/(\triangle CO_2 + \triangle CO) \quad \text{(Eq. S2)}$$

For a pure high-temperature intensity (flaming) fire, the MCE is near 0.99, while MCEs are lower for smoldering fires, between 0.65–0.85 (22). An overall fire-integrated MCE near 0.9 indicates that roughly equal amounts of biomass are being consumed by high intensity (flaming) and smoldering processes. Combustion can transition between high temperature and smoldering types during a single event, and both can occur simultaneously in a single event. Moreover, there may be no distinct transitional state between types.

Numerous studies have shown that particulate matter emissions from fires are negatively related to their CE or MCE (20, 23-27). This is especially true for char because these particles are combustion residues produced by pyrolysis (28, 29); indeed, char emissions can be used to calculate CE (30). In comparison, the production of soot is fundamentally different because it forms primarily through gas-to-particle conversion under oxidizing conditions (18, 28, 29). Positive correlations have been found between soot production and MCE (22, 26, 31, 32). For example, Christian et al. (22) studied emissions from a variety of representative fuels, and these authors found that the emission factors for soot were positively correlated with MCE. This was also recently observed by May et al. (26) and Yokelson et al. (31), who found that the number of soot particles measured with a single particle soot photometer was positively correlated with MCE in emissions from both open fires and domestic biomass burning.

Another factor that may influence the relative proportions of char and soot in combustion emissions is the production of ash, which is sometimes referred to as char or charcoal (33). Brewer et al. (33) found that for fuels with 4 to 8% moisture, the post-fire residues averaged 0.02% while for the 13-18% moisture group, the residues were higher, 0.05%. This means that more char is produced for wetter fuels compared with drier ones. Therefore, these studies support the idea that char is inversely related to MCE, that is, high intensity (flaming) versus smoldering combustion, while soot is positively related to MCE.

In a paleo-wildfire record from a Northeastern American lake sediment, Han et al. found more char in samples from wetter conditions, and more soot during drier climates (34). This result is consistent with studies of modern seasonal savanna carbon emissions which showed an anticorrelation between fuel moisture and combustion efficiency (35). Thus, it is reasonable to infer that more char is emitted from smoldering fires, and more soot is emitted from high intensity fires. Charcoal is another term describing the combustion residues. However, charcoal specifically refers to those particles identified with the use of a microscope in sediment studies.

A study of the spatial distribution of char and soot in aerosols from 14 Chinese cities (36) showed relatively similar soot concentrations but highly variable char. Thus, char was considered to originate locally while soot came from broader regional sources (36, 37). This pattern is not only consistent with the differences in the ways in which char and soot form, but also with their physical and chemical properties (28, 38); this is because soot particles are submicron-sized and are more readily transported than the larger char particles. The regional distribution of soot has been documented in the study of Jeong et al. (39) who found similar variations of soot concentrations in urban, rural, and village areas in Canada. Thus, in general, char (or charcoal) reflects local (or sometimes regional for char) smoldering wildfires, while soot reflects regional to continental high intensity wildfires in paleo-wildfire reconstructions.

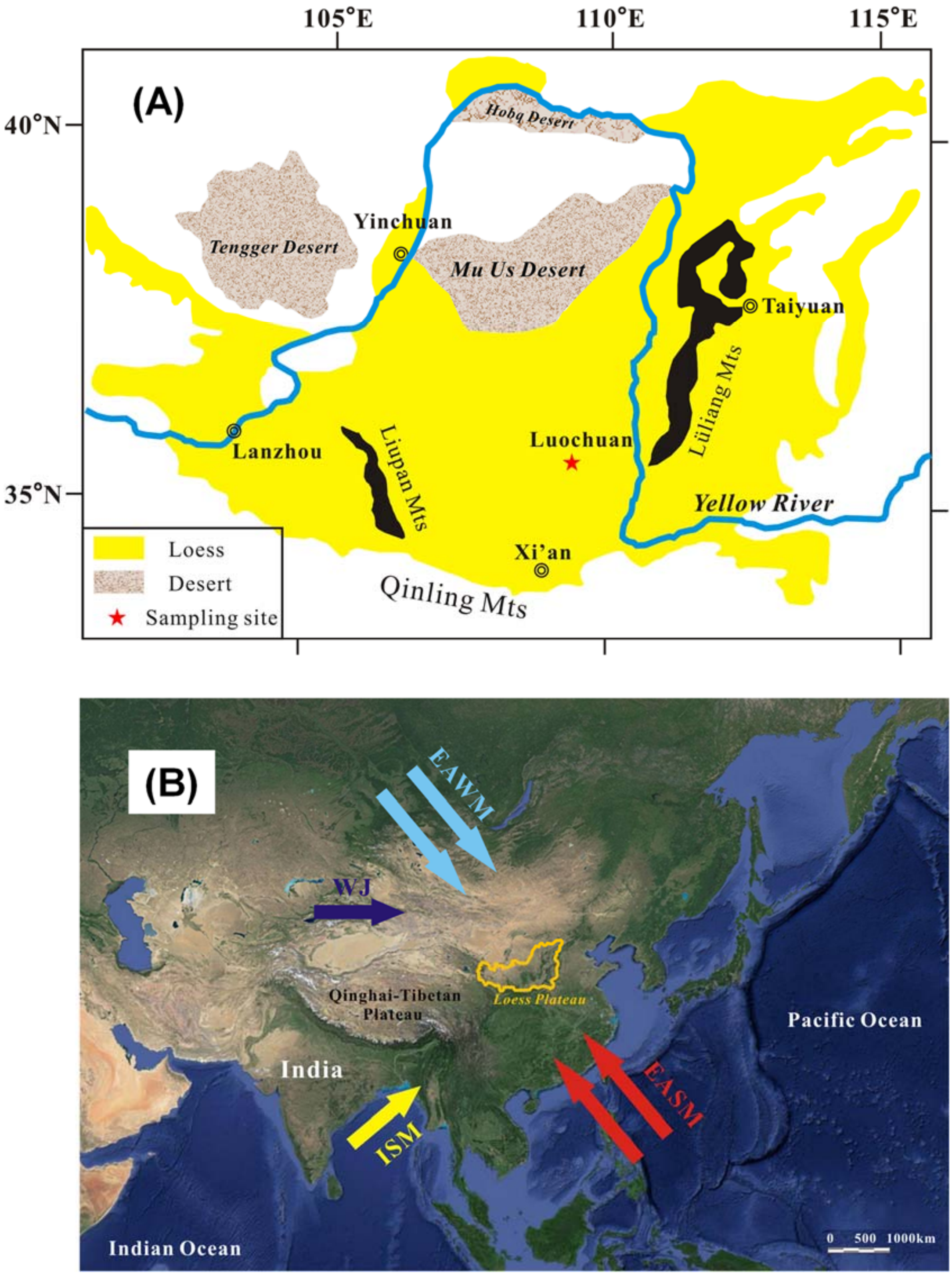

Fig. S1. Study site location and major climatic influences. (A) location of the classical Luochuan section on the Chinese Loess Plateau; (B) climatic influences on the plateau, including the East Asian summer and winter monsoons (EASM and EAWM, respectively), the Indian summer monsoon (ISM), and the Westerly jet (WJ).

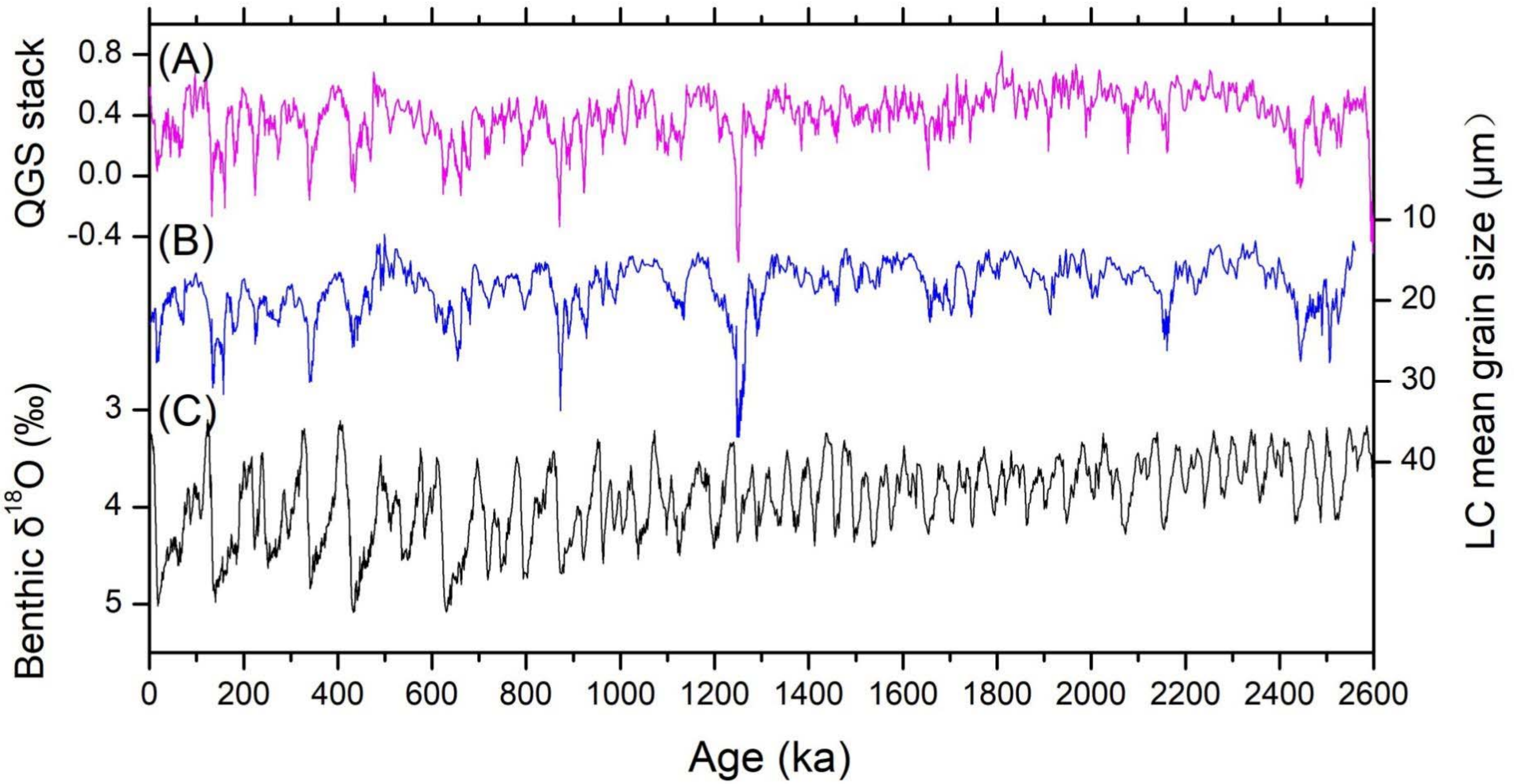

Fig. S2. Comparison of the variations of (B) Luochuan (LC) mean grain size in this study versus (A) the stacked quartz grain size (QGS) in the Chinese Loess Plateau (10, 40) and (C) the marine benthic $\delta^{18}O$ stack (42).

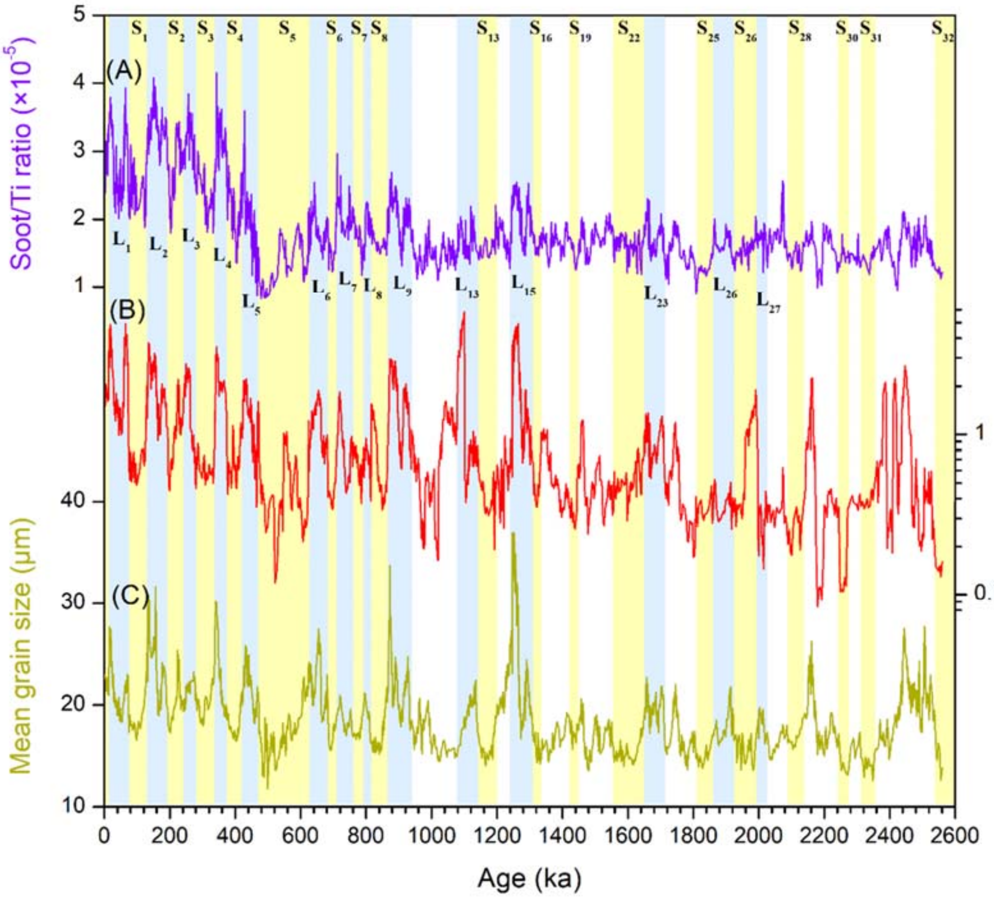

Fig. S3. Time-series variations of (A) soot concentrations normalized by Ti in comparison with (B) soot mass accumulation rates and (C) mean grain size in the Luochuan section of the Chinese Loess Plateau during the Quaternary period. The yellow shaded areas indicate paleosol (S) layers, corresponding to interglacial periods, while the light blue shaded areas indicate loess (L) layers, corresponding to glacial periods. Below L9, not all layers reflecting glacial-interglacial cycles are shown with different colors; this was done to highlight only the main patterns in the cycles.

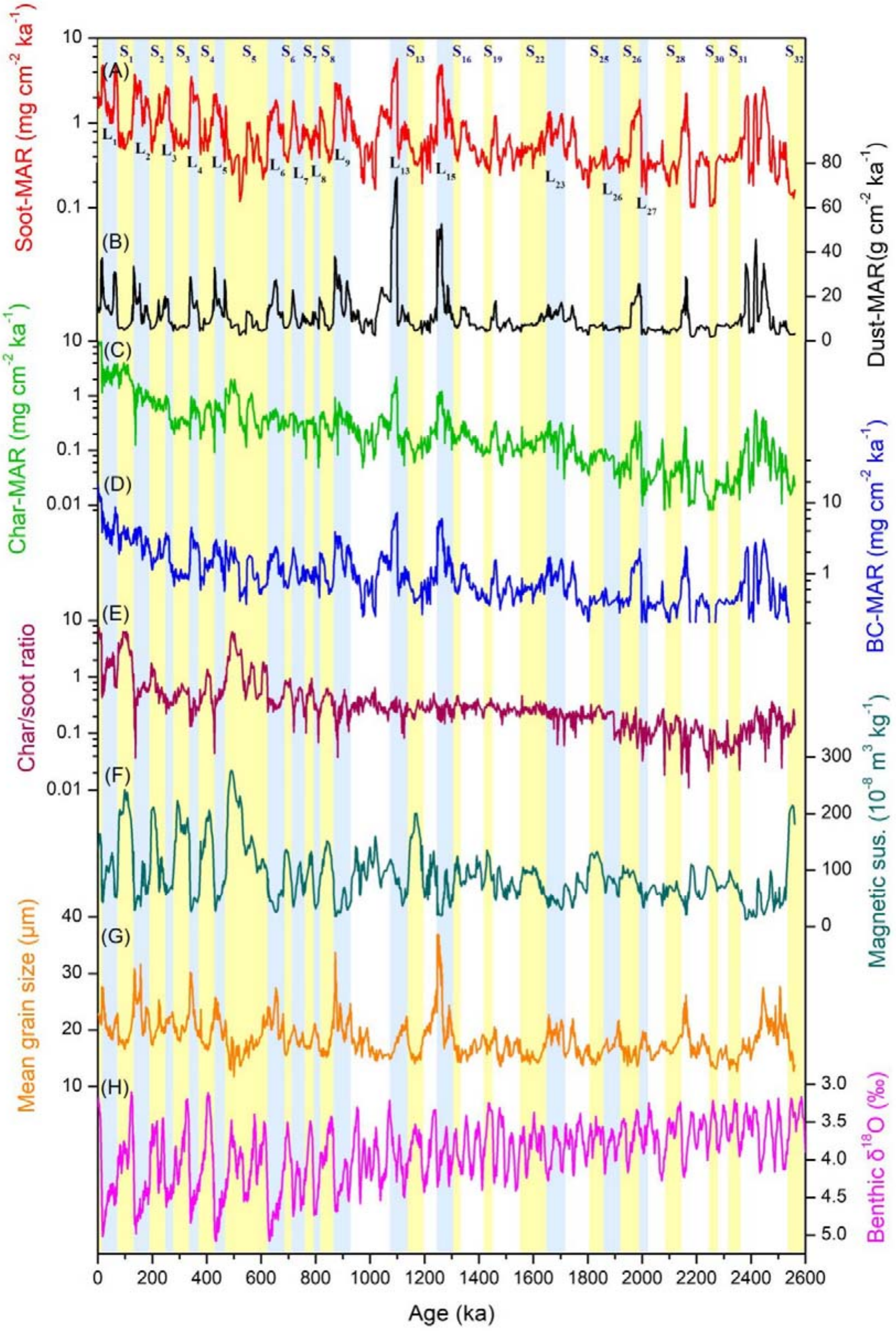

Soot-MAR (mg cm-2 ka-1)
Dust-MAR(g cm-2 ka-1)
Char-MAR (mg cm-2 ka-1)
BC-MAR (mg cm-2 ka-1)
Char/soot ratio
Magnetic sus. (10-8 m3 kg-1)
Mean grain size (μm)
Benthic δ18O (‰)
Age (ka)
(A)
(B)
(C)
(D)
(E)
(F)
(G)
(H)
S1 S2 S3 S4 S5 S6 S7 S8 S13 S16 S19 S22 S25 S26 S28 S30 S31 S32
L1 L2 L3 L4 L5 L6 L7 L8 L9 L13 L15 L23 L26 L27

Fig. S4. Wildfire history reconstructed from black carbon (BC), char, and soot mass accumulation rates (MARs) in the Luochuan section of the Chinese Loess Plateau (CLP) during the entire Quaternary period. (A) soot-MARs, indicating high intensity fires; (B) dust-MARs, indicating aridification of the loess source area (41); (C) char-MARs, indicating smoldering fires; (D) BC-MARs, indicating wildfires; (E) char/soot ratios; (F) magnetic susceptibility, an indicator of precipitation on the CLP (*9*); (G) mean grain size, an indicator of wind strength and the aridity of the dust source area (8, 41); (H) marine benthic oxygen stable isotope, an indicator of ice volume (42). The yellow and light blue shaded areas indicate paleosol (S) and loess (L) layers, respectively, as in Fig. S3.

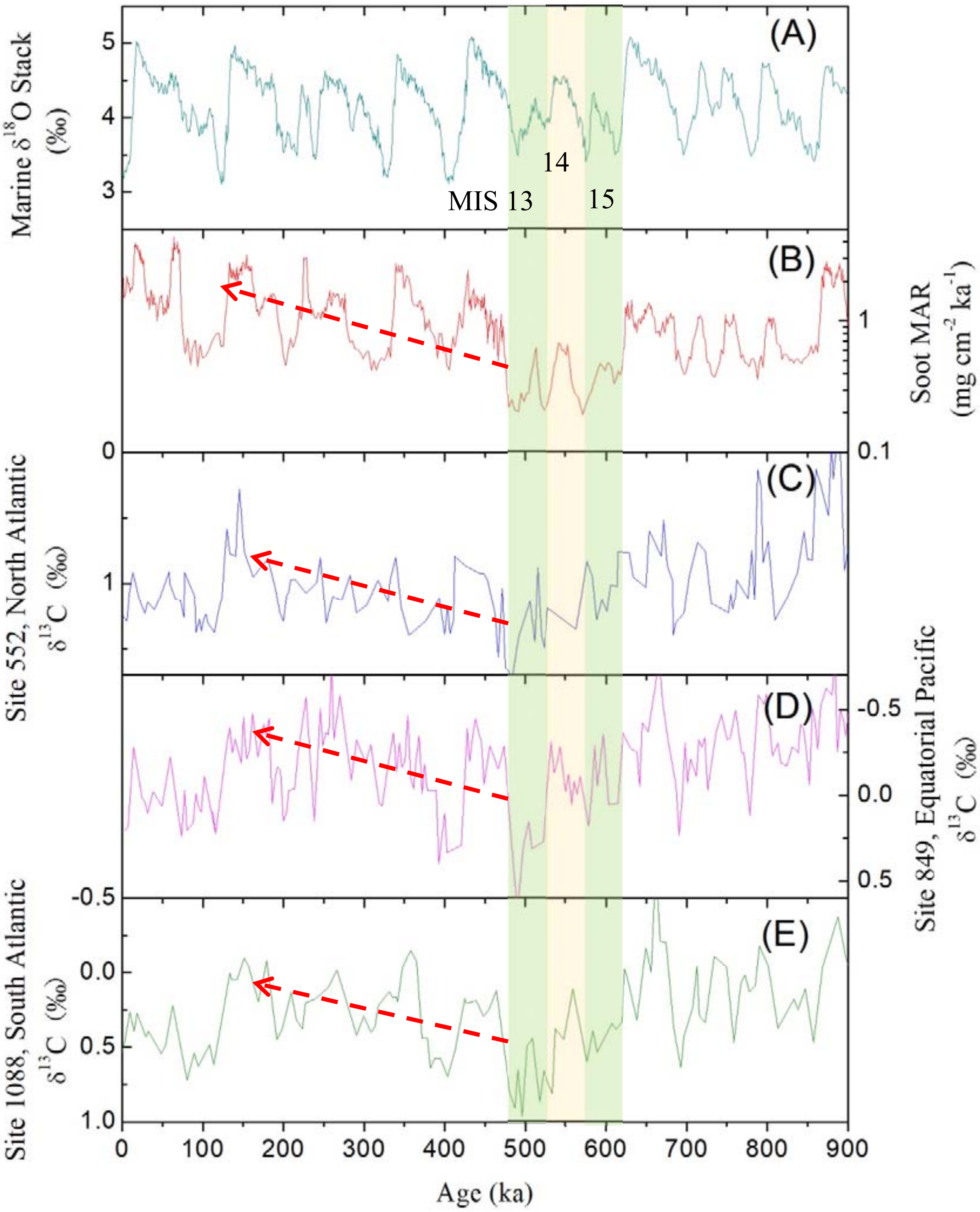

Fig. S5. The 900-ka record of soot mass accumulation rates (MARs) in the Luochuan section compared with marine benthic $\delta^{13}C$ records. (A) Marine $\delta^{18}O$ stack, an indicator of ice volume (43); (B) soot MAR; (C-E) benthic $\delta^{13}C$ components from North Atlantic (44), Equatorial Pacific, and South Atlantic (45), respectively. Note the inverse scales for the benthic $\delta^{13}C$ values; that is, the benthic $\delta^{13}C$ records show a decreasing trend over the past 500 ka (dashed red lines). The shaded area indicates marine isotope stages (MIS) 13, 14, and 15, corresponding to relatively low biomass burning: MIS 14 was a mild glacial period and MIS 13 and 15 were interglacial periods.

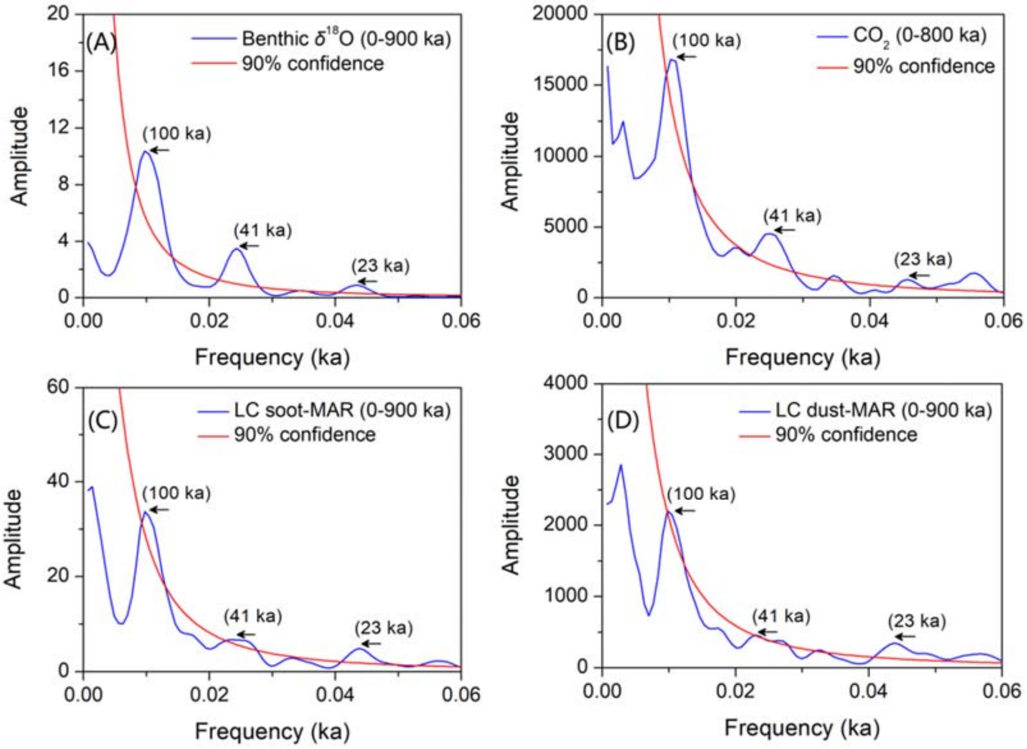

Fig. S6. Similar profiles of spectral frequency analyses for marine $\delta^{18}O$ (42) , atmospheric CO_2 (46), soot-MARs, and dust loadings from the Luochuan section in this study. Note: the time duration for atmospheric CO_2 is over the past 800 ka; while for the other three parameters it is over the past 900 ka**.**

Table S1. Principal Component Analysis (PCA, rotated component matrix) for data used in this study, with PCA loadings > 0.6 shown in bold. It produces two principal components, indicating two different combustion characteristics: high intensity fires (PC1, characterized mainly by soot MAR) and smoldering fires (PC2, characterized mainly by char concentration and char MAR). Note: All data were interpolated into a 1ka interval to facilitate the PCA analysis. Concentrations and mass accumulation rate (MAR) of BC, char, and soot were log-transformed.

Parameter	Component		Communality
	1	2	
BC concentration	0.303	**0.907**	0.915
Char concentration	0.226	**0.962**	0.976
Soot concentration	**0.630**	0.513	0.660
Char/soot ratio	0.078	**0.955**	0.918
BC-MAR	**0.742**	**0.640**	0.960
Char-MAR	0.525	**0.833**	0.969
Soot-MAR	**0.927**	0.295	0.947
Dust-MAR	**0.910**	0.121	0.843
Mean grain size	**0.817**	0.085	0.675
Magnetic Susceptibility	-0.556	**0.733**	0.846
Bulk density	**-0.758**	-0.097	0.584
[1] Marine ^{18}O	**0.715**	0.117	0.524
Initial Eigenvalue	5.12	4.90	
Percent of variance	42.7	39.1	
Cumulative percent	42.7	81.8	

[1]The data that were not generated from our analyses are the marine ^{18}O (from reference 42).

Table S2. Mass accumulation rates (MARs) for soot, char, and dust; magnetic susceptibility (MS); and mean grain size (MGS) measured in the Luochuan section of the Chinese Loess Plateau.

Age (ka)	Soot MAR (mg cm^{-2} ka^{-1})	Char MAR (mg cm^{-2} ka^{-1})	MS (10^{-8} m^3 kg^{-1})	MGS (μm)	Dust MAR (g cm^{-2} ka^{-1})
1.0	2.14	14.69	136.2	22.9	15.0
2.2	1.49	10.89	143.9	22.6	14.0
3.5	1.58	11.86	145.3	22.6	14.6
4.7	1.74	10.12	148.4	22.4	14.3
6.0	2.19	11.82	159.6	21.4	15.4
7.2	1.92	10.44	164.9	21.6	14.0
8.5	1.93	9.05	161.4	22.7	14.0
9.8	1.64	9.54	155.1	21.9	14.2
11.0	1.80	9.99	149.2	21.1	14.9
12.2	1.73	10.28	143.8	21.0	14.7
13.2	1.93	4.88	94.9	22.2	16.5
14.1	2.41	1.51	46.0	25.3	19.0
14.8	2.77	1.67	44.6	27.7	22.4
15.3	3.77	1.65	83.7	25.8	27.8
15.9	3.65	3.98	68.2	26.4	27.9
16.5	3.91	3.65	57.0	27.6	28.2
17.1	3.72	1.93	45.8	27.3	28.0
17.6	3.71	2.12	44.9	26.7	26.9
18.2	3.55	1.82	42.4	27.4	27.6
18.8	3.48	1.79	45.3	25.6	26.8
19.4	3.75	1.81	50.5	27.4	25.7
20.0	3.33	2.12	53.7	25.6	24.7
20.6	3.41	1.91	59.4	24.6	24.7
21.2	3.35	2.14	66.6	23.8	24.3
21.8	2.94	3.20	66.2	24.1	24.2
22.5	3.49	2.75	67.5	23.8	24.7
23.1	3.08	1.72	75.4	24.3	23.1
23.8	2.79	2.28	76.4	22.0	22.7
24.5	3.08	2.64	81.9	22.3	22.2
25.2	2.88	2.16	83.4	22.0	21.8
25.9	2.70	2.43	83.1	21.7	22.0
26.6	2.94	2.73	84.1	22.2	21.9
27.4	2.17	1.96	82.9	23.0	19.6

28.2	2.13	3.12	91.8	20.4	18.4
29.1	1.69	2.03	97.9	20.3	17.1
30.0	1.55	2.75	103.5	21.2	16.7
31.0	1.39	1.83	107.7	20.7	15.8
32.1	1.52	3.15	99.4	20.0	14.7
33.1	1.39	2.79	104.3	20.6	14.2
34.2	1.22	2.73	106.4	19.8	14.9
35.2	1.63	2.55	102.8	19.9	14.1
36.3	1.73	3.40	103.1	20.0	13.9
37.3	1.31	1.93	101.9	20.4	13.9
38.4	1.34	2.18	96.3	19.0	14.0
39.4	1.59	2.63	102.5	20.4	14.0
40.5	1.34	2.47	96.5	20.2	14.4
41.5	1.36	2.00	106.1	19.0	13.7
42.6	1.16	2.47	105.6	19.4	13.5
43.7	1.00	1.72	110.3	20.8	13.9
44.8	1.30	2.07	108.7	19.7	13.3
45.8	1.34	2.52	113.5	18.9	13.7
46.9	1.51	2.39	114.9	18.4	13.8
48.0	1.50	2.94	123.8	19.0	13.3
49.2	1.57	2.91	121.1	19.6	13.2
50.3	1.22	2.53	123.9	18.4	13.4
51.4	1.10	1.72	125.0	19.0	13.3
52.5	1.27	2.84	130.7	18.4	13.2
53.6	1.21	2.37	117.4	18.3	13.5
54.7	1.14	3.07	124.5	18.4	13.8
55.8	1.57	3.36	110.2	19.8	14.0
56.9	1.24	2.20	104.7	19.8	13.8
57.9	1.28	1.96	96.2	20.3	13.9
58.9	1.60	3.80	99.9	20.3	17.0
59.7	1.91	2.35	75.2	20.3	17.3
60.5	2.79	1.95	53.6	21.3	20.7
61.2	2.98	4.34	76.9	20.4	22.5
61.8	3.01	2.69	65.1	21.5	26.0
62.3	2.89	2.27	56.7	21.3	26.7
62.9	3.62	2.42	52.2	21.9	26.8
63.5	2.86	2.27	55.2	21.8	27.1
64.1	4.36	3.36	51.3	21.7	27.5
64.6	3.56	2.53	55.1	21.8	27.6
65.2	3.48	2.44	54.2	21.7	28.0
65.8	3.56	2.96	54.7	21.7	27.8

66.4	3.59	2.85	53.9	22.4	28.5
66.9	3.54	2.71	54.1	21.2	27.4
67.5	3.56	2.57	56.9	22.0	27.4
68.1	3.57	3.30	55.9	21.8	27.2
68.6	4.00	2.57	54.6	21.6	27.4
69.2	3.25	2.60	50.7	22.3	27.0
69.8	3.32	2.93	58.1	22.6	27.2
70.3	3.40	3.54	61.9	22.1	27.8
70.9	3.55	3.66	62.4	22.5	28.1
71.4	3.21	3.85	66.0	23.0	27.7
72.5	1.82	2.55	69.5	22.8	15.2
74.0	1.07	1.44	96.5	22.6	10.2
76.0	0.72	2.94	162.1	17.3	7.9
78.5	0.56	1.51	197.5	19.0	6.3
81.4	0.74	2.63	207.3	18.0	5.2
84.4	0.47	1.48	197.8	18.0	5.1
87.4	0.61	2.86	198.8	17.6	5.3
90.4	0.72	2.64	204.0	18.3	5.4
93.4	0.45	2.87	200.7	18.1	5.2
96.5	0.66	4.04	223.4	17.0	5.4
99.6	0.53	3.28	242.2	16.6	5.4
102.6	0.53	2.57	232.3	17.7	5.3
105.7	0.57	3.60	223.6	18.4	5.7
108.7	0.62	2.69	237.6	17.9	6.0
111.6	0.70	4.08	232.5	17.7	6.2
114.5	0.76	2.57	212.4	18.3	6.3
117.3	0.80	2.37	208.0	19.1	6.5
120.1	0.80	1.91	199.3	19.4	6.7
122.7	0.64	1.86	191.5	20.0	7.3
125.2	0.65	1.80	171.4	21.2	7.3
127.4	1.04	1.35	140.7	22.4	8.4
129.1	1.24	0.99	80.0	24.0	10.0
130.5	1.57	1.78	132.6	26.2	11.3
131.6	2.04	1.15	79.3	26.5	14.2
132.4	2.79	1.74	77.9	22.3	20.6
133.1	2.76	1.44	57.6	28.8	20.0
133.9	2.20	0.33	28.2	30.8	20.5
134.6	2.54	0.29	29.6	29.5	21.7
135.3	2.13	0.52	30.5	28.9	21.3
136.0	2.14	0.30	30.2	27.9	20.8
136.8	2.32	0.43	29.2	30.3	19.4

137.5	2.46	0.09	33.5	26.1	20.2
138.3	2.07	0.23	36.8	26.2	18.1
139.1	2.33	0.65	37.4	25.4	18.2
139.9	2.03	0.80	42.5	25.3	17.8
140.7	2.35	1.04	39.6	25.6	17.9
141.6	2.57	0.84	50.7	24.9	17.6
142.4	1.89	0.60	50.0	24.7	16.8
143.2	2.29	0.94	45.2	24.7	16.8
144.1	2.25	0.92	48.8	24.6	16.9
144.9	2.48	1.17	54.2	24.8	17.3
145.7	2.40	0.77	52.0	24.7	18.3
146.6	2.60	1.55	55.5	24.9	18.5
147.4	2.27	1.45	52.8	25.0	18.4
148.2	2.77	1.49	53.1	25.4	17.4
149.0	2.75	1.59	48.1	25.8	17.8
149.8	2.75	1.44	50.1	25.1	18.9
150.6	2.62	1.34	47.8	25.2	18.9
151.4	2.47	1.37	47.4	26.7	18.9
152.2	2.66	1.37	52.9	26.3	18.5
153.0	3.19	1.40	48.7	25.7	19.3
153.8	2.51	1.40	47.7	25.4	18.5
154.6	2.58	1.21	49.5	26.3	18.9
155.3	2.40	1.15	48.8	26.8	18.6
156.1	2.65	1.23	46.5	28.1	18.9
156.8	2.43	1.09	35.1	31.7	20.1
157.5	2.41	1.45	48.4	26.8	19.6
158.3	2.60	1.38	46.7	29.0	18.9
159.3	1.96	1.25	64.6	24.3	15.8
160.3	1.89	1.19	64.5	22.6	14.4
161.5	1.57	0.96	88.2	21.2	12.0
162.9	1.44	0.95	100.2	21.5	10.7
164.4	1.19	0.84	115.8	19.2	9.8
166.0	1.23	0.78	72.0	19.0	9.7
167.5	1.08	0.84	100.7	19.4	9.5
169.0	1.17	0.95	104.1	19.7	9.7
170.6	1.30	0.95	111.1	19.5	10.0
172.0	1.24	1.03	110.8	21.5	10.2
173.5	1.52	0.91	94.6	19.6	10.5
174.9	1.47	0.87	75.2	20.8	11.1
176.3	1.43	0.90	56.9	22.5	10.9
177.6	1.48	0.83	74.5	24.2	12.1

178.9	1.43	0.92	61.3	22.7	11.9
180.2	1.51	0.83	67.6	23.6	12.4
181.5	1.58	0.95	67.6	23.8	12.5
182.7	1.47	0.80	58.1	23.8	12.2
184.0	1.59	1.01	62.9	23.4	11.9
185.3	1.57	0.92	76.0	23.2	11.4
186.7	1.50	0.85	81.3	22.9	10.9
188.1	1.41	0.89	75.0	21.7	10.7
189.7	1.09	0.82	88.8	20.2	8.8
191.7	0.86	0.60	93.0	20.1	7.5
194.0	0.78	0.69	117.1	19.6	6.5
196.5	0.54	0.95	204.3	17.6	5.8
199.3	0.46	0.70	208.5	17.4	5.5
202.2	0.59	0.61	208.9	17.3	5.6
204.9	0.74	1.03	212.0	18.5	5.8
207.7	0.62	0.84	202.9	18.9	6.0
210.3	0.71	0.84	197.8	18.6	6.3
212.9	0.87	0.48	173.6	19.7	6.2
215.2	1.21	0.64	173.7	19.8	7.6
217.2	1.02	0.56	131.8	20.2	8.8
219.0	1.24	0.97	138.1	20.4	10.9
220.5	1.68	1.09	111.1	20.8	15.3
221.7	3.02	1.94	109.3	20.5	23.7
222.8	2.91	1.75	99.5	21.7	24.3
223.9	2.98	1.02	73.0	25.4	23.2
225.0	3.02	1.21	61.5	24.6	25.3
226.1	3.03	1.43	77.7	22.8	25.6
227.2	3.03	1.32	69.0	24.4	24.0
228.3	2.18	0.95	99.0	23.2	19.0
229.5	1.72	1.02	120.0	21.3	15.7
230.7	1.39	0.72	102.1	20.7	13.1
231.9	1.40	0.71	107.8	20.9	11.2
233.2	1.12	0.75	123.2	19.6	10.2
234.5	1.23	0.76	120.2	20.6	10.2
235.7	1.06	0.57	96.4	20.0	9.9
237.0	1.05	0.52	104.1	20.1	10.0
238.3	1.06	0.55	100.8	20.4	10.0
239.6	1.14	0.60	102.4	19.9	10.2
240.8	1.13	0.40	86.8	20.2	10.0
242.1	1.03	0.43	74.9	19.4	9.9
243.4	1.19	0.42	72.7	20.6	9.9

244.8	1.09	0.43	69.7	19.5	9.5
246.3	1.27	0.45	62.4	19.7	10.3
247.9	1.18	0.59	76.5	19.6	9.8
249.6	1.44	0.53	56.1	21.9	10.5
251.3	1.45	0.33	58.7	21.4	10.8
253.0	1.70	0.74	73.1	21.7	11.2
254.7	1.40	0.50	60.0	22.1	11.1
256.4	1.30	0.53	60.1	21.2	11.6
258.2	1.63	0.56	61.7	21.5	11.3
259.9	1.59	0.44	62.1	22.3	11.9
261.6	1.51	0.42	63.7	21.1	11.3
263.2	1.57	0.53	65.3	22.2	12.3
264.9	1.57	0.55	67.4	22.3	12.5
266.6	1.59	0.59	68.4	22.3	12.8
268.2	1.42	0.50	70.3	22.4	12.6
269.9	1.27	0.41	71.6	22.4	11.9
271.5	1.48	0.47	68.5	22.5	12.1
273.2	1.46	0.52	65.7	23.2	12.7
274.8	1.16	0.42	77.9	22.3	11.6
276.5	1.59	0.51	88.4	22.8	12.3
278.5	0.93	0.45	104.3	20.5	9.2
280.8	0.78	0.34	119.0	21.2	7.5
283.4	0.68	0.38	152.1	19.5	6.9
286.3	0.71	0.24	150.4	20.6	5.7
289.5	0.61	0.23	197.8	20.3	5.2
292.8	0.61	0.29	223.3	18.5	5.2
296.2	0.57	0.37	216.4	18.2	5.0
299.6	0.51	0.23	212.6	18.7	4.9
302.9	0.58	0.33	192.9	18.0	5.2
306.1	0.59	0.32	168.2	19.1	5.2
309.3	0.42	0.32	180.4	20.8	5.5
312.4	0.50	0.36	177.2	20.8	5.3
315.5	0.42	0.28	164.0	20.5	5.6
318.6	0.48	0.27	181.2	19.5	5.5
321.6	0.52	0.30	162.8	19.9	5.6
324.7	0.50	0.28	176.6	20.5	5.5
327.6	0.54	0.27	183.2	21.7	5.7
330.4	0.63	0.36	193.1	22.6	6.1
332.9	0.52	0.34	166.2	22.0	7.0
334.9	0.98	0.36	165.7	23.2	8.6
336.5	1.11	0.32	141.3	24.6	10.9

337.7	1.60	0.32	89.3	27.3	14.2
338.5	2.25	0.30	71.7	28.0	21.3
339.3	2.74	0.44	37.2	30.2	21.5
340.0	2.44	0.41	32.6	29.3	21.2
340.8	2.34	0.63	26.3	29.4	21.2
341.5	2.39	0.32	28.9	29.3	21.6
342.3	2.40	0.42	26.4	29.9	21.2
343.1	2.67	0.75	27.4	30.0	21.3
343.9	2.37	0.32	29.8	27.5	21.2
344.6	2.39	0.44	30.1	27.7	20.4
345.5	2.39	0.62	32.7	28.0	19.8
346.3	2.55	0.63	33.1	26.9	19.7
347.2	2.67	0.44	36.9	25.6	18.9
348.1	2.27	0.52	39.0	25.7	18.1
349.0	1.85	0.70	43.6	23.5	17.1
349.9	1.75	0.54	45.1	23.2	17.1
350.9	2.04	0.88	46.4	22.6	16.6
351.9	2.03	0.76	50.1	22.5	16.5
352.9	2.23	0.89	48.0	22.6	16.0
353.9	2.20	0.76	51.4	22.2	16.0
354.9	2.16	0.76	49.8	23.6	16.2
355.9	2.12	0.84	50.1	21.0	16.1
359.3	1.87	0.52	49.9	22.4	14.7
362.7	1.83	0.56	48.6	20.2	12.8
365.9	1.43	0.30	46.3	21.4	12.2
369.1	1.35	0.32	44.8	20.2	11.6
372.2	1.30	0.35	44.2	19.9	11.3
372.8	1.32	0.41	44.4	20.1	10.9
373.4	1.33	0.36	42.4	19.8	11.2
374.1	1.27	0.35	43.6	19.0	10.7
374.7	1.39	0.40	50.4	19.2	10.7
375.4	1.42	0.38	51.4	18.9	10.5
376.0	1.20	0.31	53.5	19.9	10.2
376.7	1.28	0.41	57.0	20.0	10.2
377.3	1.19	0.39	85.5	18.6	10.6
378.0	0.83	0.50	96.4	18.6	10.5
378.7	0.97	0.37	151.1	19.0	10.0
379.6	0.89	0.30	121.6	23.6	9.5
380.9	1.02	0.38	118.9	18.1	8.6
382.5	0.84	0.35	122.7	17.7	8.2
384.5	0.81	0.37	129.2	17.9	7.7

386.7	0.77	0.39	130.5	17.9	7.1
389.0	0.69	0.35	151.1	17.4	7.0
391.3	0.52	0.29	154.8	17.3	6.9
393.7	0.81	0.39	140.3	16.8	7.2
396.0	0.79	0.48	168.7	16.7	7.2
398.3	0.58	0.59	190.2	17.5	7.0
400.7	0.62	0.69	191.8	17.1	7.4
403.1	0.45	0.62	194.1	16.5	7.3
405.4	0.43	0.56	187.0	17.0	7.0
407.7	0.58	0.60	187.2	16.5	7.1
410.0	0.76	0.86	206.4	17.7	8.0
412.3	0.63	0.62	201.2	17.3	8.3
414.4	0.75	0.79	198.5	18.8	8.7
416.4	0.96	0.66	188.3	19.3	9.1
418.4	1.16	0.40	170.7	20.5	9.6
420.4	0.94	0.38	162.5	21.2	9.7
422.3	0.88	0.25	153.0	19.9	10.4
424.0	1.34	0.32	128.3	21.3	11.0
425.5	1.60	0.32	95.3	22.4	13.6
426.7	1.77	0.27	71.3	23.8	13.8
427.8	2.13	0.15	77.3	22.7	18.8
428.7	2.43	0.11	56.7	21.8	17.9
429.6	1.93	0.46	42.3	23.9	20.0
430.5	1.30	0.42	28.6	25.8	20.1
431.4	1.47	0.70	37.8	24.9	21.9
432.2	1.20	0.37	27.1	24.9	18.9
433.1	1.29	0.40	31.6	23.7	19.5
433.9	1.56	0.55	32.8	25.7	20.6
434.8	1.60	0.54	41.8	24.1	20.6
435.7	1.75	0.62	41.6	22.5	18.9
436.7	1.47	0.50	43.0	22.1	17.7
437.6	1.24	0.51	47.6	22.3	17.8
438.6	1.30	0.52	48.5	21.8	17.3
439.5	1.56	0.64	55.5	22.1	17.7
440.5	1.47	0.57	54.2	22.1	17.2
441.4	1.32	0.55	52.1	24.9	17.9
442.3	1.46	0.65	53.8	21.9	17.8
443.3	1.44	0.56	50.3	21.9	17.4
444.2	1.43	0.65	49.8	22.0	17.5
445.2	1.19	0.75	50.1	22.1	17.8
446.1	1.52	0.63	46.1	22.8	17.2

447.1	1.24	0.56	52.7	22.9	17.9
448.0	1.26	0.55	49.3	22.0	18.0
449.0	1.58	0.64	53.6	21.8	17.2
450.0	1.03	0.55	52.6	21.4	17.0
451.0	1.22	0.52	53.9	20.9	16.0
452.0	1.12	0.48	55.9	20.7	15.0
453.0	0.67	0.40	56.1	20.5	15.4
454.1	0.96	0.50	59.0	19.2	15.1
455.1	0.84	0.49	59.3	20.1	14.8
456.2	1.19	0.57	62.2	18.8	14.4
457.3	1.04	0.55	70.3	18.3	14.0
458.4	0.94	0.44	74.6	19.6	13.7
459.5	1.02	0.54	72.4	18.2	13.7
460.5	0.61	0.31	69.9	18.5	14.5
461.6	0.96	0.58	72.8	19.7	14.6
462.7	1.01	0.61	80.8	18.8	14.6
463.8	1.05	0.64	79.4	19.3	14.8
464.8	0.60	0.50	78.0	18.8	15.6
465.8	0.50	0.30	75.3	20.3	16.2
466.8	0.64	0.55	83.3	20.5	17.6
467.8	0.86	0.66	82.4	21.8	17.9
468.8	0.91	0.93	86.8	20.4	18.8
469.8	1.12	0.95	88.2	21.2	18.3
470.8	0.70	0.78	95.8	20.4	17.6
472.1	0.91	0.76	107.7	20.1	14.7
473.7	0.58	0.86	163.9	17.2	12.3
475.6	0.51	0.82	179.9	17.4	11.0
477.9	0.31	0.72	229.7	16.3	7.2
480.5	0.22	0.42	246.2	16.1	5.8
483.2	0.25	0.50	256.5	13.1	4.8
485.9	0.21	0.78	269.3	14.3	5.0
488.6	0.20	1.03	276.3	12.9	4.8
491.1	0.28	1.66	275.5	18.8	5.6
493.8	0.24	1.49	274.0	15.5	5.7
496.4	0.27	1.36	266.9	18.1	6.4
499.3	0.28	1.20	258.5	11.8	6.5
502.5	0.38	2.22	248.5	14.3	7.4
505.9	0.43	1.51	234.2	15.1	7.7
509.3	0.52	1.49	221.3	17.1	9.4
513.1	0.61	1.46	221.3	15.4	10.6
516.9	0.40	1.01	210.9	17.3	7.2

520.9	0.23	0.56	231.2	13.6	5.0
524.9	0.21	0.64	221.5	13.7	4.0
528.9	0.23	0.44	207.7	14.6	4.2
532.7	0.30	0.26	160.9	14.8	4.2
536.4	0.45	0.26	143.8	15.6	5.0
539.9	0.51	0.44	144.2	16.8	6.2
543.2	0.67	0.27	126.7	15.8	8.0
546.6	0.66	0.30	127.4	18.2	8.1
549.9	0.59	0.34	133.7	16.2	7.8
553.3	0.59	0.41	142.0	17.0	8.1
556.6	0.54	0.56	144.4	15.1	8.1
559.8	0.65	0.49	138.7	17.9	8.3
563.0	0.51	0.67	149.8	17.6	8.3
566.1	0.34	0.62	160.4	19.1	6.3
569.2	0.28	0.40	155.2	18.8	4.8
572.3	0.26	0.40	149.8	16.7	4.3
575.6	0.19	0.29	141.1	16.5	3.4
578.8	0.24	0.19	136.9	17.4	3.4
582.0	0.29	0.17	124.9	17.2	3.8
585.2	0.34	0.14	113.1	18.3	4.2
588.4	0.41	0.16	95.5	17.1	4.9
591.5	0.47	0.20	101.1	17.5	5.7
594.6	0.44	0.23	107.7	17.8	5.7
597.6	0.41	0.19	103.9	18.8	5.9
600.6	0.47	0.24	101.0	18.7	5.9
603.4	0.47	0.23	112.3	18.9	6.1
606.2	0.43	0.52	119.5	19.3	6.8
608.9	0.33	0.61	119.8	22.0	6.8
611.5	0.35	0.64	114.1	22.7	7.0
614.1	0.41	0.50	104.2	20.4	7.1
616.7	0.38	0.56	109.5	20.9	7.3
619.1	0.42	0.69	102.7	21.8	8.1
621.1	0.59	0.58	95.8	20.9	9.3
622.8	0.64	0.33	71.9	23.5	11.0
624.2	1.27	0.31	57.7	24.0	13.6
625.3	1.21	0.52	84.9	24.2	18.3
626.4	1.09	0.30	51.1	24.0	17.8
627.5	1.40	0.54	63.8	22.3	17.0
628.6	1.11	0.47	55.5	23.7	17.2
629.7	1.02	0.41	53.4	23.8	16.6
630.8	1.12	0.42	44.7	24.0	17.1

632.0	1.15	0.48	50.2	22.0	16.4
633.1	1.45	0.53	46.0	22.9	16.5
634.3	1.04	0.41	46.3	20.8	14.8
635.6	1.02	0.33	45.9	22.1	13.7
636.8	0.88	0.33	43.2	20.0	12.9
638.0	0.97	0.33	45.2	21.7	12.3
639.3	0.81	0.33	44.5	20.0	11.9
640.5	0.98	0.35	43.9	21.4	11.8
641.8	1.04	0.35	42.7	20.7	11.7
643.0	0.97	0.36	43.0	20.8	12.2
644.2	0.95	0.38	39.0	21.2	12.7
645.4	1.10	0.27	37.1	21.1	13.6
646.6	1.02	0.39	36.4	22.5	14.6
647.7	1.04	0.29	34.1	23.0	16.2
648.8	1.21	0.42	30.4	23.6	17.6
649.9	1.22	0.36	29.3	24.3	18.0
651.0	1.13	0.31	27.9	24.5	18.5
652.0	1.26	0.38	26.1	25.5	18.9
653.0	1.27	0.39	26.8	26.2	19.0
654.0	1.29	0.43	26.1	25.7	19.7
655.0	1.36	0.40	25.4	27.5	19.1
655.9	1.33	0.36	25.6	26.9	19.5
656.9	1.30	0.38	25.8	26.9	19.9
657.9	1.22	0.33	28.7	25.0	19.3
658.9	1.30	0.39	28.2	25.2	19.3
660.0	1.10	0.33	27.4	26.0	17.1
661.0	1.09	0.31	29.3	24.8	16.3
662.0	1.06	0.34	35.7	20.8	15.1
663.0	1.00	0.30	46.4	19.6	13.7
664.0	0.80	0.23	55.8	18.3	12.9
665.1	0.82	0.27	58.4	18.9	12.5
666.1	0.81	0.23	56.2	18.7	12.3
667.2	0.84	0.28	56.7	18.5	12.3
668.3	0.75	0.27	57.0	18.8	12.7
669.3	0.81	0.24	56.3	18.7	12.4
670.4	0.88	0.32	56.7	19.3	12.6
671.4	0.99	0.31	54.2	18.5	13.0
672.4	0.89	0.31	58.1	19.9	13.0
673.4	0.80	0.30	63.6	20.0	12.6
674.4	0.97	0.37	63.6	19.5	13.0
675.4	1.07	0.46	64.4	20.5	14.1

676.3	0.94	0.43	62.2	20.7	14.4
677.3	1.05	0.44	59.9	21.3	14.0
678.2	1.08	0.50	58.3	20.7	14.2
679.2	1.15	0.49	59.8	19.7	13.9
680.1	1.05	0.44	53.6	21.5	14.2
681.1	1.06	0.47	53.0	23.0	13.5
682.0	1.09	0.46	66.5	20.4	13.8
683.4	0.77	0.37	96.7	20.5	10.9
685.2	0.50	0.29	99.8	18.6	9.0
687.4	0.61	0.47	130.7	16.1	8.1
690.0	0.53	0.46	134.9	15.6	7.3
693.0	0.45	0.33	136.1	15.6	6.6
695.9	0.40	0.30	130.8	16.1	6.9
698.8	0.40	0.33	132.5	16.5	7.0
701.6	0.46	0.30	123.7	18.1	6.9
704.3	0.54	0.40	115.7	17.5	7.5
706.9	0.51	0.48	104.7	17.9	7.7
709.4	0.69	0.40	85.0	19.0	8.4
711.7	0.77	0.40	75.8	19.0	9.4
713.9	0.82	0.34	56.1	19.6	10.3
716.0	0.88	0.28	40.6	19.6	11.2
717.9	1.07	0.29	34.7	20.0	13.2
719.9	1.05	0.11	35.9	21.0	12.7
721.8	1.10	0.34	46.2	20.4	12.9
723.8	1.06	0.32	49.6	19.9	12.2
725.9	1.00	0.45	64.3	19.2	12.4
728.0	0.76	0.30	74.7	18.6	9.2
730.1	0.71	0.28	74.4	18.2	7.9
732.3	0.49	0.20	85.9	17.4	6.5
734.6	0.48	0.18	92.1	18.1	5.7
736.8	0.39	0.24	105.7	17.3	5.0
739.1	0.38	0.23	107.1	17.0	5.1
741.3	0.43	0.28	111.1	17.4	5.3
743.5	0.48	0.32	114.5	18.3	6.0
745.7	0.61	0.37	103.0	18.2	7.2
747.7	0.56	0.30	77.4	17.9	8.1
749.6	0.72	0.43	92.4	18.8	10.2
751.4	1.12	0.44	64.4	19.6	12.8
753.1	0.96	0.34	58.4	18.8	12.7
754.8	1.00	0.35	54.2	18.5	12.9
756.4	0.94	0.19	60.2	17.3	12.5

758.1	0.90	0.23	78.4	17.0	11.9
759.9	0.96	0.26	60.1	17.1	12.0
761.6	0.95	0.25	62.9	16.9	11.9
763.4	1.14	0.35	75.9	17.3	12.0
765.1	1.02	0.10	80.8	17.0	11.7
766.8	0.96	0.47	83.8	17.1	10.0
768.6	0.75	0.26	90.4	16.6	8.9
770.3	0.66	0.29	94.2	16.9	7.7
772.1	0.52	0.27	98.3	17.2	7.1
773.8	0.55	0.27	103.2	17.3	6.7
775.5	0.48	0.24	105.2	17.0	6.3
777.3	0.48	0.29	117.3	17.2	6.4
779.0	0.45	0.34	128.7	16.7	6.6
780.7	0.51	0.29	122.7	17.3	6.5
782.4	0.52	0.24	109.1	16.8	6.4
784.1	0.47	0.35	133.1	18.1	6.9
785.8	0.36	0.34	127.2	17.6	7.0
787.4	0.49	0.29	125.0	18.9	7.3
788.9	0.55	0.30	98.2	18.9	7.4
790.5	0.44	0.27	89.4	19.3	8.2
792.2	0.56	0.28	67.4	19.7	8.9
793.9	0.82	0.25	54.5	20.3	10.8
795.7	0.95	0.25	37.1	21.2	12.7
797.5	0.97	0.27	37.6	21.1	14.2
799.4	1.06	0.36	30.6	20.2	14.5
801.4	0.97	0.30	34.0	19.5	13.5
803.3	1.04	0.27	37.3	19.1	13.0
805.4	0.87	0.24	37.9	18.6	13.3
807.4	1.04	0.27	41.1	19.3	13.1
809.6	1.05	0.07	45.2	18.7	12.7
811.7	0.95	0.23	69.1	16.8	10.8
814.0	0.68	0.13	71.1	16.2	9.4
816.2	0.66	0.19	83.9	16.4	8.4
818.5	0.65	0.15	91.9	16.4	7.6
820.8	0.51	0.18	96.2	15.4	7.0
823.1	0.56	0.20	93.0	16.6	7.0
825.4	0.56	0.22	110.3	15.7	6.9
827.7	0.53	0.20	119.0	15.3	7.1
830.1	0.50	0.24	107.3	16.8	6.7
832.4	0.51	0.23	125.2	15.4	6.9
834.7	0.48	0.22	132.3	15.7	7.1

837.0	0.51	0.25	140.7	16.1	6.9
839.3	0.52	0.27	134.3	16.3	7.2
841.7	0.49	0.31	141.8	15.2	7.1
844.0	0.47	0.29	151.5	16.2	7.0
846.3	0.52	0.32	149.7	15.4	7.2
848.6	0.52	0.25	148.8	15.7	7.4
850.8	0.54	0.28	146.9	16.8	7.5
852.9	0.57	0.28	144.6	18.4	7.8
854.9	0.65	0.34	136.0	17.1	8.3
856.9	0.56	0.30	126.0	18.8	8.5
858.8	0.60	0.29	126.1	18.8	8.7
860.7	0.67	0.21	105.0	20.5	9.1
862.5	0.58	0.28	98.6	21.0	9.6
864.0	0.84	0.28	97.6	21.8	11.0
865.3	1.01	0.34	81.7	20.3	12.6
866.4	1.31	0.27	55.1	22.1	15.7
867.3	1.56	0.44	50.7	23.9	19.2
867.9	2.18	0.65	47.0	24.2	27.5
868.5	2.18	0.77	45.5	23.9	28.5
869.1	1.89	0.43	32.5	26.4	29.0
869.6	1.77	0.58	29.7	26.5	29.0
870.2	2.08	0.48	31.3	26.6	29.9
870.7	2.58	0.98	29.4	26.5	32.4
871.2	2.57	0.81	17.9	30.2	33.2
871.7	2.49	0.47	18.4	33.7	33.8
872.2	2.48	0.54	19.5	31.4	34.7
872.7	2.42	0.50	20.4	29.9	34.1
873.2	2.60	0.29	20.6	28.5	31.9
873.7	2.84	0.53	21.0	28.7	31.4
874.2	2.40	0.34	18.7	28.4	30.3
874.8	2.39	0.38	18.1	28.3	30.0
875.3	2.52	0.33	21.4	25.5	29.1
875.9	2.55	0.23	21.9	25.0	27.7
876.5	2.49	0.36	21.3	25.1	26.3
877.2	2.27	0.39	22.2	23.0	25.4
877.8	2.57	0.42	24.0	23.2	25.0
878.5	2.21	0.17	25.1	21.8	23.7
879.2	2.35	0.36	26.8	20.0	23.2
879.9	1.93	0.07	26.0	21.1	22.7
880.6	1.89	0.28	26.4	19.8	21.8
881.4	1.87	0.22	27.8	20.7	22.1

882.1	1.79	0.31	28.2	20.0	22.2
882.8	1.86	0.48	27.4	20.2	21.6
883.6	1.95	0.50	27.5	20.0	22.9
884.3	1.93	0.46	26.2	21.0	22.6
885.0	2.13	0.62	25.0	21.6	23.0
885.7	2.32	0.66	24.7	21.9	23.5
886.4	2.14	0.66	24.0	20.7	24.5
887.0	2.20	0.63	24.3	21.8	24.3
887.7	2.26	0.48	24.0	21.8	25.9
888.3	2.56	0.39	23.3	24.1	26.4
889.0	2.51	0.63	24.2	23.8	26.1
889.6	2.76	0.52	24.4	24.7	27.6
890.2	2.43	0.46	24.4	23.8	26.6
890.8	2.48	0.57	24.4	24.2	26.3
891.4	2.31	0.47	29.6	24.0	26.1
892.0	2.00	0.60	24.7	23.8	25.8
892.7	2.07	0.66	28.3	23.8	25.6
893.3	2.48	0.46	29.0	22.6	25.1
894.0	2.31	0.60	32.8	22.9	24.4
894.6	2.04	0.63	35.0	22.6	24.8
895.4	1.76	0.47	31.7	22.4	21.4
896.3	1.47	0.51	45.6	20.5	17.9
897.3	1.41	0.30	48.0	20.3	15.7
898.4	1.08	0.39	50.7	19.7	14.4
899.6	1.04	0.36	54.8	20.1	13.0
900.9	0.88	0.38	62.3	19.9	12.9
902.2	0.85	0.41	67.5	18.9	13.0
903.5	0.88	0.39	60.9	18.3	12.4
904.8	0.81	0.38	63.8	19.0	12.4
906.1	0.70	0.42	69.7	17.9	12.3
907.3	0.81	0.38	64.4	19.3	12.7
908.6	0.66	0.32	65.4	19.3	12.8
909.9	0.71	0.34	58.0	19.6	13.0
911.1	0.96	0.35	57.5	19.6	13.2
912.3	0.72	0.33	49.2	18.4	13.2
913.5	0.91	0.32	36.1	21.2	13.8
914.7	1.18	0.32	34.8	21.1	14.3
915.8	1.03	0.28	29.5	21.0	14.7
916.8	1.13	0.17	29.3	21.2	16.1
917.8	1.24	0.17	30.3	22.3	17.0
918.7	1.35	0.21	30.0	20.9	18.3

919.6	1.31	0.30	33.4	21.7	18.6
920.4	1.58	0.40	34.0	22.0	19.1
921.3	1.67	0.38	32.8	21.6	18.7
922.1	1.58	0.17	33.9	21.7	18.9
922.9	1.31	0.32	35.6	22.2	18.7
923.8	1.60	0.30	35.1	22.9	19.6
924.6	1.51	0.24	36.4	23.0	20.2
925.4	1.72	0.43	36.0	22.9	20.0
926.2	1.70	0.31	40.0	23.8	19.8
927.0	1.50	0.40	40.2	23.3	19.4
927.7	1.47	0.37	41.1	24.7	20.7
928.5	1.58	0.31	44.5	22.2	20.4
929.3	1.53	0.34	42.8	23.4	21.1
930.3	1.48	0.40	43.9	23.1	16.8
931.4	1.28	0.33	56.9	21.7	13.9
932.7	1.05	0.35	82.6	18.7	12.9
934.1	0.99	0.31	89.3	17.5	10.8
935.6	0.86	0.25	88.8	17.9	10.1
937.2	0.71	0.24	91.3	18.2	10.0
938.7	0.81	0.22	94.8	18.5	10.2
940.3	0.71	0.25	96.4	18.0	9.8
941.9	0.77	0.19	98.3	17.6	9.8
943.5	0.69	0.20	100.1	17.9	9.4
945.2	0.65	0.23	113.2	16.7	9.7
946.8	0.61	0.18	141.3	15.7	9.6
948.5	0.63	0.18	113.8	17.0	9.4
950.3	0.59	0.17	124.4	16.5	9.0
951.9	0.64	0.16	138.2	16.2	10.1
953.6	0.49	0.18	126.0	16.8	9.8
955.2	0.49	0.22	98.4	17.8	10.4
956.8	0.55	0.14	115.2	17.4	10.6
958.4	0.59	0.13	112.3	17.2	10.7
960.0	0.58	0.20	98.6	17.9	11.1
961.6	0.75	0.34	60.3	20.3	11.0
963.1	0.65	0.22	54.4	20.6	11.5
964.8	0.41	0.17	59.4	19.4	7.9
966.6	0.36	0.11	75.0	18.6	6.4
968.4	0.26	0.11	108.2	16.3	5.1
970.3	0.23	0.08	110.0	15.2	4.5
972.1	0.30	0.08	99.8	16.7	3.9
974.0	0.22	0.05	105.9	18.6	3.8

975.8	0.31	0.09	100.4	17.5	4.0
977.5	0.27	0.09	114.8	17.8	4.6
979.2	0.30	0.11	101.1	18.6	5.1
980.9	0.33	0.12	94.1	18.5	6.0
982.6	0.44	0.16	89.5	18.7	7.2
984.2	0.52	0.24	86.8	19.4	9.2
985.8	0.51	0.21	85.5	19.7	9.4
987.4	0.56	0.23	81.7	19.8	9.6
989.0	0.55	0.22	86.1	20.4	9.1
990.7	0.55	0.23	93.0	19.7	8.6
992.4	0.42	0.16	103.1	18.0	7.7
994.1	0.38	0.14	119.5	18.7	6.9
996.0	0.30	0.11	124.9	16.3	6.5
997.9	0.33	0.12	133.7	16.5	6.1
999.8	0.33	0.13	140.7	15.2	6.6
1001.8	0.44	0.14	133.9	15.9	7.2
1003.7	0.46	0.20	112.1	16.3	8.4
1005.6	0.61	0.25	115.2	17.1	9.9
1007.5	0.77	0.41	111.0	17.1	11.9
1009.4	0.75	0.40	104.2	16.8	12.0
1011.3	0.79	0.38	111.7	15.7	11.5
1013.3	0.79	0.24	114.3	15.9	10.9
1015.3	0.73	0.30	126.5	15.8	10.5
1017.4	0.72	0.47	151.4	14.9	10.5
1019.5	0.75	0.37	159.7	14.6	10.9
1021.6	0.65	0.20	151.3	15.0	10.7
1023.8	0.67	0.18	143.1	16.4	10.6
1025.9	0.69	0.18	129.2	14.5	10.9
1027.9	0.73	0.16	114.2	15.0	10.8
1030.1	0.57	0.20	109.9	15.6	11.0
1032.4	0.70	0.26	90.9	15.8	11.7
1034.9	0.89	0.31	87.7	16.4	12.5
1037.5	0.77	0.26	73.4	16.5	12.8
1040.3	0.96	0.24	68.8	16.2	13.3
1043.1	0.87	0.26	85.5	15.5	13.0
1045.9	0.76	0.22	86.8	14.9	13.1
1048.8	0.69	0.16	93.6	15.3	12.5
1051.7	0.84	0.25	98.2	15.7	12.6
1054.6	0.72	0.21	99.2	15.6	12.4
1057.4	0.67	0.15	98.1	15.5	12.1
1060.3	0.82	0.20	99.0	15.5	11.5

1063.1	0.81	0.19	102.9	15.7	11.2
1066.0	0.53	0.09	107.9	15.7	10.3
1068.8	0.62	0.17	110.1	15.8	9.8
1071.7	0.65	0.19	115.1	14.8	9.9
1074.7	0.49	0.13	118.5	15.3	10.0
1077.6	0.62	0.22	117.2	15.0	9.8
1080.4	0.57	0.21	109.8	15.6	10.0
1083.1	0.66	0.18	111.4	16.0	10.1
1085.5	0.58	0.22	103.4	16.1	10.3
1087.8	0.81	0.20	96.0	16.1	10.4
1089.8	0.61	0.21	94.4	16.8	10.6
1091.7	0.72	0.29	90.5	17.4	10.7
1093.5	0.73	0.18	82.5	17.7	10.9
1095.3	0.76	0.23	75.7	17.8	10.6
1097.1	0.81	0.37	85.4	17.6	10.8
1098.8	0.79	0.19	74.4	17.9	10.6
1100.6	0.85	0.21	74.5	18.9	10.7
1102.4	0.74	0.23	75.9	18.0	10.7
1104.1	0.80	0.24	82.9	17.8	10.9
1105.8	0.62	0.19	86.8	18.7	11.0
1107.5	0.76	0.23	82.3	18.6	11.5
1109.2	0.65	0.26	67.3	19.5	11.6
1110.8	0.82	0.25	68.5	19.5	11.4
1112.3	0.67	0.20	67.6	20.0	11.9
1113.8	1.02	0.20	58.1	20.1	11.9
1115.1	0.70	0.18	56.5	19.0	12.3
1116.4	0.64	0.09	46.4	20.9	12.2
1117.5	0.70	0.07	40.5	20.0	12.4
1118.6	0.76	0.08	38.2	20.8	12.6
1119.8	0.71	0.08	36.2	21.0	12.4
1120.9	0.65	0.14	39.4	19.8	12.7
1122.1	0.68	0.07	40.9	20.4	11.9
1123.2	0.71	0.15	41.7	20.5	11.5
1124.4	0.61	0.14	42.8	19.9	11.2
1125.6	0.57	0.05	45.4	20.0	11.3
1126.7	0.64	0.15	50.8	20.4	11.4
1127.9	1.01	0.22	48.2	20.7	11.8
1129.0	0.90	0.27	67.4	20.8	12.3
1130.1	1.04	0.27	53.6	20.1	12.1
1131.2	0.77	0.21	52.0	21.9	12.4
1132.3	0.77	0.21	52.8	20.1	12.7

1133.4	1.09	0.22	51.4	22.2	13.0
1134.5	0.88	0.31	55.1	22.4	12.3
1135.6	0.98	0.23	58.2	21.6	12.3
1137.1	0.74	0.26	56.7	19.0	11.7
1138.9	0.81	0.20	66.4	19.2	11.1
1141.1	0.58	0.15	86.5	17.4	8.6
1143.6	0.47	0.13	123.1	17.0	7.2
1146.4	0.43	0.12	149.8	16.2	6.3
1149.4	0.37	0.10	159.8	15.8	5.5
1152.4	0.30	0.07	156.0	14.8	4.9
1155.4	0.35	0.07	164.3	15.8	4.9
1158.6	0.31	0.07	164.8	15.7	4.9
1161.7	0.32	0.06	172.8	14.7	5.0
1164.8	0.35	0.07	200.7	14.0	5.0
1168.0	0.37	0.10	199.6	15.3	4.9
1171.2	0.36	0.10	201.1	14.9	4.8
1174.3	0.31	0.10	189.1	14.6	4.7
1177.3	0.30	0.12	178.2	15.5	4.7
1180.4	0.30	0.10	173.5	15.1	4.7
1183.4	0.32	0.09	159.6	16.1	4.7
1186.3	0.29	0.09	154.2	14.7	4.7
1189.3	0.34	0.10	99.2	17.3	5.2
1192.2	0.35	0.13	142.6	17.2	5.7
1195.1	0.54	0.14	69.4	18.9	6.4
1198.0	0.55	0.15	69.0	18.7	7.4
1201.0	0.66	0.17	50.9	20.1	9.0
1203.9	0.60	0.14	53.6	19.0	8.6
1206.8	0.57	0.14	65.1	19.5	8.9
1209.6	0.53	0.17	90.8	20.8	9.1
1212.5	0.70	0.18	46.9	20.6	9.0
1215.3	0.71	0.20	101.0	19.7	9.5
1217.9	0.69	0.16	56.7	21.3	9.3
1220.4	0.63	0.26	127.1	19.2	8.5
1222.7	0.70	0.22	102.2	20.0	8.4
1224.7	0.66	0.18	89.1	21.3	8.1
1226.5	0.58	0.17	111.2	21.4	8.0
1228.3	0.53	0.17	103.3	23.3	8.1
1230.0	0.61	0.18	112.4	21.6	8.4
1231.7	0.57	0.21	117.0	23.4	8.5
1233.4	0.55	0.24	131.4	24.0	8.8
1235.1	0.49	0.26	133.5	23.1	8.8

1236.7	0.60	0.23	112.3	22.9	8.7
1238.3	0.55	0.21	101.8	23.4	8.9
1239.9	0.46	0.15	91.5	25.9	9.0
1241.4	0.61	0.22	72.7	25.7	9.5
1242.9	0.43	0.12	77.6	26.4	9.5
1244.2	0.69	0.23	71.9	27.2	10.1
1245.4	0.71	0.21	107.2	23.1	11.9
1246.3	1.10	0.35	41.6	29.2	15.3
1247.0	1.40	0.39	19.6	36.9	19.3
1247.6	2.11	0.40	24.7	35.0	26.9
1248.0	2.26	0.44	22.0	34.9	29.6
1248.5	2.14	0.43	40.3	32.2	29.8
1249.0	2.14	0.50	21.4	36.9	30.2
1249.5	2.40	0.50	20.1	36.2	28.7
1250.0	2.27	0.60	21.4	35.6	30.2
1250.5	2.57	0.69	29.3	36.9	30.0
1250.9	2.18	0.66	21.2	35.1	28.8
1251.4	2.35	0.45	22.5	33.3	29.6
1251.9	2.57	0.45	20.8	36.8	28.8
1252.4	2.63	0.63	20.8	35.4	28.8
1252.9	2.17	0.63	22.2	32.7	28.1
1253.4	2.76	0.62	21.4	33.0	27.8
1253.9	2.30	0.49	20.7	31.1	27.3
1254.5	2.35	0.70	20.5	34.0	28.0
1255.0	2.43	0.49	20.2	34.4	27.6
1255.5	2.70	0.47	19.9	34.9	29.1
1256.0	2.49	0.58	20.6	32.1	28.4
1256.5	2.52	0.41	20.3	31.3	27.9
1257.1	2.37	0.45	19.9	30.9	26.2
1257.6	2.35	0.47	20.7	33.0	25.9
1258.2	2.32	0.39	19.3	30.8	26.2
1258.7	2.05	0.57	19.4	30.1	25.6
1259.3	2.35	0.53	19.8	30.6	25.6
1259.9	2.40	0.64	20.7	27.8	25.5
1260.4	1.95	0.50	19.8	32.0	26.7
1261.0	2.44	0.39	19.5	31.7	26.0
1261.6	2.46	0.46	20.1	31.6	25.5
1262.1	2.37	0.60	19.2	28.7	26.0
1262.7	1.88	0.60	19.2	28.7	24.2
1263.3	2.31	0.49	19.1	29.0	24.8
1263.9	2.18	0.51	20.0	29.9	23.4

1264.6	2.04	0.37	21.3	25.6	21.6
1265.3	2.06	0.46	20.7	24.7	22.0
1265.9	1.90	0.38	30.4	22.5	20.9
1266.6	2.07	0.36	36.3	22.8	20.2
1267.3	1.83	0.41	38.1	22.2	19.7
1268.0	2.12	0.46	38.5	19.7	20.3
1268.6	1.13	0.31	43.0	21.0	18.8
1269.3	1.84	0.38	51.4	20.6	20.0
1270.1	1.77	0.41	46.6	18.9	18.8
1270.8	1.36	0.42	49.5	20.5	17.9
1271.6	1.49	0.36	60.8	17.8	17.1
1272.4	1.13	0.38	59.3	16.7	17.2
1273.2	1.50	0.38	57.5	17.6	16.4
1274.0	1.22	0.36	70.0	17.4	16.3
1274.8	1.03	0.15	82.6	17.5	16.8
1275.6	1.07	0.30	94.1	17.5	16.5
1276.4	1.10	0.33	92.3	17.7	17.3
1277.1	1.18	0.36	97.2	17.6	17.9
1277.9	1.26	0.40	98.0	18.4	18.0
1278.7	0.89	0.31	93.8	18.5	17.7
1279.5	1.23	0.48	89.4	18.4	18.1
1280.2	1.24	0.37	77.0	17.9	18.2
1281.0	1.15	0.44	98.9	18.3	18.1
1281.7	1.08	0.41	101.7	19.2	18.2
1282.5	1.20	0.51	95.3	19.3	18.6
1283.2	1.12	0.44	89.8	20.0	19.4
1283.9	1.31	0.51	73.0	19.5	19.7
1284.5	1.35	0.47	70.7	20.3	20.3
1285.2	1.24	0.45	64.3	21.5	19.6
1285.9	1.46	0.44	49.6	22.3	20.1
1286.6	1.70	0.58	76.5	18.5	20.3
1287.4	1.87	0.54	60.1	19.9	20.3
1288.2	1.67	0.38	62.7	20.8	20.8
1289.1	1.77	0.47	53.5	22.3	22.2
1290.1	1.80	0.42	43.7	24.4	22.1
1291.1	1.98	0.49	38.0	23.1	22.1
1292.1	2.18	0.60	36.3	22.3	22.9
1293.1	1.86	0.37	34.3	23.6	22.1
1294.2	1.71	0.55	33.6	23.0	21.4
1295.3	1.77	0.43	35.7	21.2	21.2
1296.4	1.82	0.43	42.6	21.8	21.5

1297.5	1.77	0.48	46.8	19.8	20.0
1298.7	1.71	0.44	48.1	21.3	19.7
1299.9	1.28	0.20	55.3	19.1	19.4
1301.1	1.45	0.35	56.7	19.6	19.0
1302.3	1.73	0.40	44.2	19.9	19.0
1303.5	1.36	0.38	46.7	19.0	19.2
1304.9	1.28	0.35	58.8	19.3	15.0
1306.4	0.92	0.23	74.7	19.2	11.8
1308.1	0.70	0.13	76.7	18.4	9.5
1309.9	0.63	0.19	84.3	16.6	8.2
1311.8	0.46	0.16	110.7	16.4	6.9
1313.9	0.46	0.15	98.1	16.9	6.6
1315.9	0.40	0.13	104.1	17.3	6.6
1318.1	0.41	0.19	120.3	15.5	6.6
1320.2	0.44	0.21	124.8	14.9	7.1
1322.4	0.44	0.21	118.5	14.3	7.6
1324.6	0.53	0.21	93.7	17.2	8.0
1326.8	0.59	0.22	109.4	14.6	9.3
1329.0	0.71	0.23	98.8	15.8	10.5
1331.3	0.68	0.22	75.7	14.2	10.5
1333.5	0.77	0.24	75.6	14.4	10.4
1335.7	0.68	0.19	71.2	15.0	10.2
1337.8	0.68	0.16	78.1	16.6	10.9
1340.0	0.75	0.17	78.7	15.6	11.3
1342.1	0.83	0.27	77.9	15.8	11.4
1344.2	0.65	0.23	81.3	15.9	9.9
1346.2	0.60	0.11	75.3	16.1	9.1
1348.3	0.52	0.18	74.7	16.2	8.2
1350.4	0.51	0.16	81.3	16.5	7.6
1352.5	0.50	0.13	79.3	16.1	6.9
1354.7	0.41	0.10	105.5	15.2	7.0
1356.9	0.41	0.14	105.1	14.4	6.8
1359.1	0.38	0.12	110.6	14.8	6.9
1361.4	0.40	0.11	101.8	15.7	7.0
1363.5	0.43	0.13	95.5	15.4	6.9
1365.7	0.56	0.17	89.6	15.4	7.4
1367.8	0.48	0.15	85.2	15.6	7.8
1369.9	0.55	0.17	91.2	17.2	7.7
1371.9	0.53	0.17	85.0	16.4	8.2
1374.1	0.54	0.14	86.4	15.6	8.6
1376.4	0.57	0.16	88.6	16.7	8.8

1378.8	0.69	0.22	89.1	17.5	8.9
1381.3	0.62	0.22	90.5	18.1	9.1
1384.0	0.61	0.16	87.6	18.4	8.6
1386.7	0.50	0.19	86.9	17.9	7.5
1389.5	0.45	0.10	96.9	17.5	6.9
1392.3	0.43	0.14	125.5	15.9	6.4
1395.2	0.38	0.16	101.9	16.5	5.6
1398.1	0.32	0.11	109.2	16.6	5.7
1401.0	0.36	0.11	107.3	16.5	5.7
1403.8	0.38	0.12	115.2	16.8	6.0
1406.5	0.37	0.10	102.4	16.9	6.6
1409.2	0.47	0.13	92.8	18.0	6.9
1411.8	0.59	0.16	65.2	18.7	7.7
1414.3	0.58	0.11	69.9	19.2	8.5
1416.9	0.74	0.14	71.8	18.8	9.5
1419.5	0.53	0.12	77.1	19.0	7.9
1422.1	0.45	0.13	82.1	18.1	6.6
1424.8	0.40	0.12	98.7	17.2	5.6
1427.5	0.37	0.11	107.0	18.5	5.1
1430.3	0.30	0.10	135.6	15.7	4.6
1433.1	0.33	0.11	130.7	17.4	4.6
1436.0	0.27	0.09	125.2	16.9	4.5
1438.8	0.27	0.10	124.6	17.1	5.0
1441.5	0.31	0.09	115.7	16.3	5.4
1444.3	0.31	0.13	92.9	18.1	5.6
1447.0	0.38	0.14	81.2	17.7	6.5
1449.6	0.46	0.14	82.3	17.6	7.2
1452.2	0.41	0.13	87.6	18.8	7.1
1454.8	0.51	0.17	92.2	18.5	7.5
1457.2	0.54	0.15	51.7	20.6	7.3
1459.8	0.54	0.14	49.6	18.1	7.5
1462.4	0.48	0.14	46.3	20.1	7.1
1465.1	0.53	0.13	55.4	17.5	6.8
1467.7	0.47	0.16	92.8	15.3	6.6
1470.3	0.39	0.11	103.6	14.7	6.5
1472.9	0.40	0.12	104.0	15.9	6.5
1475.4	0.44	0.12	106.7	14.0	6.2
1477.9	0.36	0.10	96.8	13.6	6.2
1480.5	0.39	0.08	84.1	13.6	6.3
1483.0	0.38	0.10	77.6	15.6	6.4
1485.5	0.41	0.06	67.0	14.6	6.7

1487.9	0.46	0.09	66.0	14.4	7.4
1490.3	0.48	0.12	53.2	15.9	7.4
1492.7	0.46	0.13	52.5	16.0	8.2
1494.9	0.52	0.11	50.3	17.0	8.8
1497.1	0.59	0.15	68.3	17.3	8.8
1499.2	0.62	0.13	61.4	18.8	9.0
1501.3	0.64	0.16	47.4	18.7	9.0
1503.3	0.65	0.18	47.4	19.0	9.1
1505.5	0.64	0.22	73.7	17.0	9.0
1507.8	0.59	0.20	70.6	18.5	8.3
1510.3	0.51	0.12	68.4	15.5	7.0
1513.0	0.47	0.12	61.3	17.9	6.5
1515.9	0.39	0.11	80.4	15.2	5.9
1518.9	0.37	0.10	79.7	15.4	5.6
1522.0	0.36	0.09	88.0	15.5	5.4
1525.0	0.38	0.10	73.5	15.8	6.0
1527.9	0.39	0.11	73.2	16.6	6.3
1530.7	0.47	0.14	66.2	17.8	6.8
1533.4	0.52	0.14	61.9	17.7	7.1
1536.1	0.59	0.15	64.2	17.5	7.7
1538.8	0.55	0.11	49.3	18.3	7.5
1541.6	0.59	0.15	49.7	18.6	7.7
1544.4	0.55	0.14	63.2	16.5	7.5
1547.2	0.59	0.13	58.8	15.9	6.9
1550.1	0.53	0.10	55.3	18.3	6.5
1552.9	0.47	0.13	80.9	16.0	6.3
1555.5	0.49	0.11	80.9	15.9	6.0
1558.0	0.44	0.21	90.0	14.4	5.6
1560.4	0.33	0.09	93.7	15.1	5.5
1562.6	0.34	0.10	105.4	14.7	5.4
1564.7	0.40	0.09	101.4	15.5	5.6
1566.9	0.39	0.10	100.2	14.5	6.1
1569.0	0.48	0.14	92.5	15.1	6.8
1571.2	0.52	0.14	97.6	15.3	7.2
1573.3	0.52	0.16	99.7	14.9	8.3
1575.4	0.57	0.15	94.6	15.4	9.2
1577.5	0.58	0.17	99.5	16.6	9.2
1579.7	0.67	0.18	96.8	14.5	9.3
1581.8	0.67	0.16	97.1	14.6	9.4
1584.0	0.62	0.18	95.4	15.6	8.7
1586.2	0.63	0.18	97.4	14.9	9.0

1588.4	0.60	0.13	97.5	14.0	8.8
1590.5	0.60	0.14	93.4	15.0	9.0
1592.7	0.60	0.13	97.2	15.0	9.2
1594.8	0.66	0.17	92.4	15.4	8.9
1596.9	0.58	0.13	92.6	16.2	8.5
1599.0	0.36	0.11	107.0	15.2	8.2
1601.1	0.47	0.12	101.5	15.5	7.6
1603.3	0.40	0.11	99.0	14.7	7.7
1605.4	0.41	0.13	98.4	14.9	7.6
1607.6	0.53	0.13	100.4	14.8	7.7
1609.8	0.49	0.11	103.5	14.8	7.6
1612.0	0.49	0.18	90.7	14.7	7.6
1614.3	0.47	0.15	97.9	14.2	7.4
1616.5	0.41	0.14	85.1	14.6	7.2
1618.8	0.44	0.14	87.9	14.2	7.2
1621.0	0.47	0.13	81.0	14.6	7.2
1623.2	0.46	0.12	74.6	14.5	7.6
1625.4	0.49	0.12	68.1	15.0	7.9
1627.5	0.37	0.14	82.1	15.1	8.1
1629.6	0.55	0.13	61.9	15.4	8.5
1631.7	0.51	0.15	58.4	16.0	8.5
1633.7	0.57	0.12	57.2	16.1	8.6
1635.7	0.53	0.16	65.0	16.3	8.8
1637.6	0.48	0.14	71.0	16.4	9.5
1639.5	0.49	0.17	65.6	17.7	9.4
1641.4	0.41	0.09	57.8	17.0	9.4
1643.3	0.59	0.19	54.3	16.9	9.5
1645.1	0.63	0.20	50.7	17.8	10.0
1646.8	0.67	0.18	52.8	17.6	10.4
1648.5	0.72	0.20	49.9	18.8	10.9
1650.1	0.85	0.16	43.7	18.7	11.3
1651.6	0.90	0.22	39.0	19.7	12.1
1653.1	0.87	0.19	50.7	18.7	12.5
1654.5	1.01	0.25	34.0	21.8	12.8
1655.9	1.03	0.26	33.4	21.9	12.5
1657.3	1.05	0.20	35.7	22.7	13.2
1658.7	1.08	0.22	51.6	19.3	13.3
1660.1	1.02	0.19	40.2	22.1	12.3
1661.5	1.11	0.29	36.6	20.3	12.1
1663.0	0.94	0.23	44.8	20.0	11.8
1664.6	0.66	0.15	56.5	19.3	11.7

1666.1	1.15	0.20	46.2	17.2	11.3
1667.7	0.71	0.16	47.6	19.9	11.1
1669.2	0.62	0.17	47.3	19.7	11.2
1670.8	0.62	0.16	54.1	18.0	11.5
1672.3	0.78	0.21	51.1	19.3	11.7
1673.8	0.65	0.18	49.1	19.6	11.4
1675.4	0.80	0.10	47.9	19.1	12.2
1676.9	0.69	0.20	42.0	20.2	11.9
1678.4	0.71	0.17	43.9	19.1	12.0
1679.8	0.76	0.19	45.3	19.8	12.0
1681.3	0.79	0.20	41.3	20.3	12.1
1682.7	0.82	0.17	45.2	20.2	12.6
1684.2	0.66	0.18	38.1	20.5	11.1
1685.7	0.63	0.12	44.5	21.2	10.6
1687.2	0.57	0.12	47.8	18.3	9.7
1688.8	0.58	0.04	47.6	18.7	9.4
1690.4	0.54	0.08	45.5	18.0	8.3
1692.0	0.57	0.06	43.2	19.4	8.4
1693.5	0.63	0.12	38.9	17.3	9.2
1695.1	0.69	0.14	39.4	19.4	9.7
1696.6	0.71	0.15	34.4	20.1	10.1
1698.1	0.77	0.22	34.0	19.8	11.6
1699.5	0.89	0.24	35.1	20.0	12.8
1700.9	0.94	0.24	31.4	20.9	12.6
1702.3	0.90	0.22	30.2	21.8	12.5
1703.7	0.93	0.24	29.2	21.8	12.5
1705.1	1.03	0.20	32.0	21.2	12.6
1706.5	0.76	0.16	32.4	20.6	12.6
1707.9	0.96	0.21	36.2	21.0	12.0
1709.7	0.69	0.15	36.6	20.3	10.1
1711.6	0.57	0.11	56.3	17.5	9.3
1713.8	0.43	0.03	71.6	15.6	7.9
1716.2	0.32	0.06	72.7	15.9	7.1
1718.8	0.39	0.06	75.7	15.1	6.6
1721.4	0.36	0.08	72.0	15.9	6.5
1724.0	0.27	0.05	70.8	15.8	6.6
1726.5	0.40	0.08	70.0	15.9	6.9
1729.0	0.41	0.09	63.0	16.2	7.0
1731.4	0.42	0.08	66.1	16.7	7.2
1733.5	0.46	0.11	58.7	17.4	8.1
1735.4	0.58	0.11	52.9	18.5	9.1

1737.1	0.60	0.12	48.9	18.5	10.6
1738.5	0.75	0.10	45.4	20.4	11.9
1739.8	1.04	0.13	43.5	19.3	13.5
1741.0	1.01	0.23	37.1	20.6	14.6
1742.2	0.92	0.19	34.2	21.0	14.2
1743.4	1.01	0.18	32.5	20.5	14.2
1744.5	1.09	0.27	33.0	22.0	14.1
1745.7	1.05	0.12	35.8	20.0	13.8
1746.9	0.95	0.20	36.9	21.7	13.7
1748.2	0.95	0.22	45.4	18.8	13.4
1749.4	0.93	0.22	49.8	19.1	13.5
1751.0	0.74	0.15	53.6	19.0	10.8
1752.9	0.65	0.12	47.1	19.5	8.6
1755.2	0.55	0.05	53.7	17.4	7.2
1757.8	0.40	0.10	81.2	15.4	6.5
1760.7	0.34	0.07	95.4	15.0	5.7
1763.7	0.35	0.08	93.7	17.0	5.7
1766.7	0.35	0.07	77.9	15.5	5.6
1769.7	0.35	0.07	81.6	16.4	5.4
1772.8	0.32	0.05	78.3	15.3	5.5
1776.0	0.27	0.08	77.3	14.1	5.4
1779.2	0.25	0.04	78.4	15.1	5.2
1782.3	0.29	0.09	71.5	13.7	5.6
1785.4	0.29	0.06	66.6	15.6	6.0
1788.4	0.42	0.08	65.4	15.8	6.6
1791.3	0.46	0.10	64.1	16.6	7.3
1794.2	0.44	0.09	58.9	16.3	8.0
1797.2	0.56	0.10	64.9	17.4	9.0
1800.4	0.47	0.11	106.1	15.4	7.2
1803.8	0.37	0.07	97.1	17.4	6.0
1807.5	0.30	0.05	125.2	14.8	5.1
1811.5	0.18	0.06	128.5	15.2	4.6
1815.5	0.22	0.03	129.6	13.9	4.1
1819.6	0.22	0.05	126.2	15.0	4.0
1823.7	0.21	0.05	120.8	14.6	4.0
1827.8	0.20	0.04	125.4	13.8	4.1
1831.9	0.20	0.06	131.9	14.2	4.2
1835.9	0.22	0.06	132.4	14.9	4.2
1839.9	0.21	0.07	125.6	16.1	4.2
1843.7	0.22	0.07	120.4	14.6	4.2
1847.5	0.22	0.05	114.2	15.3	4.5

1851.2	0.27	0.07	112.2	15.8	4.7
1854.7	0.30	0.06	92.5	16.5	5.3
1858.1	0.35	0.08	87.0	17.1	5.9
1861.4	0.46	0.08	78.8	16.9	6.8
1864.5	0.61	0.12	59.2	17.6	8.2
1867.6	0.61	0.11	62.1	17.7	8.3
1870.8	0.47	0.11	77.6	18.5	7.0
1874.0	0.41	0.09	87.0	17.2	6.3
1877.2	0.34	0.08	90.2	16.0	5.6
1880.5	0.32	0.07	89.4	16.4	5.1
1883.9	0.28	0.07	86.8	16.4	4.7
1887.2	0.30	0.07	83.9	16.8	4.8
1890.4	0.30	0.08	83.2	16.6	4.9
1893.6	0.29	0.07	76.6	17.5	4.8
1896.8	0.34	0.08	86.2	17.4	5.3
1899.9	0.41	0.03	80.3	16.9	5.6
1902.9	0.41	0.04	75.2	18.6	6.1
1905.8	0.46	0.04	73.9	18.5	6.5
1908.5	0.49	0.05	54.0	19.6	7.0
1911.3	0.45	0.05	61.2	21.4	7.0
1914.1	0.54	0.06	52.1	21.8	7.0
1916.9	0.43	0.03	87.0	17.0	6.9
1919.8	0.40	0.03	84.4	19.7	6.4
1922.7	0.32	0.06	88.1	16.5	6.0
1925.8	0.36	0.05	84.8	16.9	5.7
1928.9	0.30	0.07	108.2	17.1	5.6
1932.1	0.35	0.03	93.8	14.4	5.6
1935.2	0.34	0.06	97.6	16.8	5.4
1938.6	0.33	0.04	94.0	17.0	5.4
1941.8	0.35	0.08	95.9	15.4	5.6
1945.0	0.33	0.05	102.3	13.7	5.6
1948.4	0.27	0.04	94.6	17.2	5.1
1951.8	0.35	0.04	87.1	15.9	5.0
1955.2	0.34	0.03	90.5	14.3	5.2
1958.6	0.29	0.04	95.4	14.2	5.1
1962.0	0.33	0.05	95.9	14.7	5.4
1965.4	0.36	0.04	99.3	15.5	5.3
1968.6	0.28	0.06	95.2	16.6	5.4
1971.8	0.38	0.05	90.9	15.5	5.4
1975.2	0.33	0.10	83.4	17.2	5.5
1978.6	0.35	0.03	77.7	14.4	5.1

1982.0	0.31	0.02	76.6	13.6	5.0
1985.2	0.33	0.03	62.7	14.2	5.5
1988.1	0.39	0.05	70.0	15.8	5.8
1990.6	0.48	0.09	58.1	16.5	6.5
1992.6	0.52	0.06	74.0	17.2	7.7
1994.4	0.59	0.11	78.3	17.5	9.5
1996.2	0.64	0.12	77.6	17.7	10.5
1997.9	0.79	0.05	51.4	18.6	11.2
1999.6	0.80	0.10	63.1	16.7	13.1
2001.2	1.00	0.03	41.5	19.4	14.5
2002.9	0.90	0.04	34.2	19.7	12.7
2004.5	0.79	0.05	35.3	19.2	12.0
2006.1	0.75	0.10	32.6	19.1	10.8
2007.8	0.78	0.07	41.7	18.3	10.4
2009.4	0.65	0.05	58.5	18.1	9.6
2011.1	0.61	0.04	48.7	19.4	9.4
2012.7	0.65	0.09	55.1	19.6	9.4
2014.4	0.47	0.07	61.9	18.4	9.4
2016.6	0.58	0.04	46.2	17.7	7.4
2019.1	0.50	0.05	50.7	16.2	6.5
2022.1	0.42	0.04	57.5	16.6	5.7
2025.6	0.24	0.03	61.1	17.3	4.8
2029.5	0.30	0.02	66.0	16.3	4.3
2033.6	0.30	0.02	68.4	14.6	4.3
2037.7	0.24	0.02	70.3	14.9	4.3
2041.9	0.27	0.02	73.5	14.6	4.2
2046.0	0.30	0.03	73.3	15.7	4.3
2050.0	0.28	0.04	69.3	15.3	4.3
2054.0	0.30	0.03	60.6	16.0	4.3
2057.9	0.31	0.05	56.5	16.2	4.3
2061.7	0.33	0.07	58.5	15.8	5.0
2065.5	0.37	0.05	59.8	16.7	5.6
2069.2	0.43	0.06	58.6	17.4	6.1
2072.8	0.47	0.09	53.9	17.3	7.1
2076.4	0.78	0.20	36.1	18.1	8.2
2080.0	0.74	0.21	76.1	16.7	8.4
2083.7	0.42	0.01	46.8	16.9	6.8
2087.4	0.36	0.06	53.4	18.0	5.7
2091.1	0.33	0.04	71.2	16.3	5.0
2094.9	0.26	0.03	52.4	16.6	4.6
2098.7	0.23	0.02	69.4	16.3	4.0

2102.6	0.22	0.01	72.5	16.4	4.1
2106.5	0.21	0.03	69.8	15.7	4.1
2110.3	0.25	0.04	70.7	16.1	4.2
2114.2	0.25	0.02	64.8	15.9	4.2
2118.0	0.27	0.03	62.5	17.0	4.2
2121.7	0.22	0.03	62.5	16.8	4.3
2125.3	0.27	0.05	68.9	17.1	4.3
2128.9	0.20	0.04	80.5	17.7	4.4
2132.3	0.25	0.04	66.8	17.7	4.5
2135.7	0.28	0.06	64.2	18.7	4.5
2139.1	0.28	0.03	52.6	18.8	4.6
2142.4	0.31	0.05	54.6	19.2	4.7
2145.4	0.35	0.01	45.6	18.7	5.4
2147.9	0.39	0.02	47.8	19.3	6.2
2150.2	0.49	0.03	45.7	21.6	7.6
2152.0	0.62	0.06	36.9	23.9	9.4
2153.4	0.94	0.12	40.0	22.3	12.9
2154.9	0.86	0.05	37.9	24.8	13.0
2156.4	0.97	0.18	31.7	24.0	13.0
2157.8	0.91	0.13	53.0	21.1	13.0
2159.3	0.94	0.08	20.2	22.3	12.1
2160.7	0.81	0.12	21.6	26.2	12.3
2162.2	0.99	0.02	20.8	21.9	12.2
2163.7	0.93	0.03	21.4	24.0	12.0
2165.3	0.90	0.04	46.6	21.4	12.1
2166.9	0.80	0.03	43.9	21.0	11.6
2168.6	0.77	0.07	60.5	19.2	11.2
2170.7	0.49	0.01	48.8	18.7	7.1
2173.4	0.38	0.03	55.6	20.4	5.8
2176.5	0.33	0.02	79.9	18.0	5.2
2180.1	0.20	0.03	110.1	15.2	4.5
2184.1	0.23	0.03	108.2	17.4	4.1
2188.3	0.26	0.03	106.1	15.7	4.8
2192.5	0.22	0.03	94.9	17.0	5.5
2196.7	0.27	0.05	64.1	18.4	6.1
2200.9	0.44	0.08	58.2	19.0	6.8
2205.0	0.34	0.04	52.1	20.8	6.1
2209.1	0.29	0.04	63.5	20.7	5.0
2213.0	0.28	0.02	48.9	17.8	4.2
2216.9	0.22	0.02	47.8	16.2	3.4
2220.6	0.21	0.03	49.0	16.3	3.0

2224.3	0.19	0.01	54.3	15.1	3.0
2228.1	0.23	0.01	59.0	16.3	3.5
2232.0	0.26	0.01	67.5	18.9	4.0
2235.9	0.29	0.01	92.4	18.6	4.8
2240.0	0.28	0.01	106.4	18.8	5.4
2244.3	0.34	0.01	106.1	17.1	5.6
2248.8	0.29	0.03	102.3	15.0	5.1
2253.3	0.27	0.02	100.9	13.6	4.6
2258.1	0.22	0.01	96.8	14.3	4.3
2263.0	0.24	0.03	89.1	13.7	4.1
2267.9	0.20	0.02	83.9	13.4	4.0
2272.6	0.22	0.02	76.9	13.1	3.9
2277.3	0.19	0.01	62.2	13.9	4.0
2281.7	0.25	0.02	70.9	15.8	4.3
2286.0	0.25	0.01	67.9	16.4	4.3
2290.2	0.25	0.02	73.2	16.7	4.3
2294.4	0.23	0.02	70.2	14.6	4.2
2298.5	0.22	0.01	70.7	14.7	4.0
2302.8	0.19	0.01	68.2	14.8	3.8
2307.0	0.22	0.01	72.7	14.3	3.9
2311.5	0.20	0.03	79.1	15.9	4.0
2316.0	0.22	0.01	78.6	15.7	4.0
2320.6	0.25	0.01	99.4	15.9	4.1
2325.3	0.24	0.01	91.4	15.9	4.0
2330.1	0.23	0.01	83.0	15.8	4.0
2334.7	0.19	0.01	80.9	14.8	3.8
2339.6	0.17	0.02	71.2	14.0	3.7
2344.3	0.20	0.01	67.8	14.1	3.7
2348.9	0.20	0.01	76.9	12.6	3.6
2353.4	0.21	0.02	62.6	15.2	3.9
2357.5	0.24	0.01	57.7	15.7	4.3
2361.3	0.27	0.04	45.3	15.6	4.9
2364.7	0.32	0.03	64.6	15.8	5.4
2368.0	0.38	0.03	41.6	16.3	6.3
2371.2	0.42	0.05	38.8	18.5	6.7
2374.4	0.42	0.06	36.6	16.7	6.8
2377.6	0.30	0.04	32.7	16.5	6.0
2380.9	0.27	0.03	12.3	17.3	5.3
2384.2	0.28	0.02	12.4	16.0	4.7
2387.4	0.31	0.04	13.0	15.9	4.7
2390.6	0.32	0.04	16.4	16.9	4.6

2393.9	0.28	0.05	29.8	18.6	5.0
2397.2	0.34	0.02	25.4	17.0	5.2
2400.5	0.36	0.04	25.7	14.7	5.5
2403.8	0.31	0.06	16.9	15.6	5.3
2407.2	0.31	0.03	17.4	16.7	5.3
2410.4	0.30	0.04	28.9	16.5	5.8
2413.5	0.29	0.03	21.7	17.9	5.8
2416.5	0.32	0.07	18.0	18.1	6.1
2419.5	0.30	0.07	15.4	19.1	6.3
2422.4	0.25	0.08	21.2	17.8	6.2
2425.2	0.27	0.05	80.8	19.0	6.5
2428.1	0.27	0.04	85.3	19.9	7.1
2430.8	0.45	0.09	62.4	18.5	7.8
2433.3	0.47	0.05	61.1	20.7	8.5
2435.5	0.52	0.13	59.7	22.3	9.7
2437.5	0.76	0.06	36.0	20.1	10.6
2439.2	0.78	0.16	28.1	23.9	12.1
2440.7	0.80	0.11	29.8	24.8	11.9
2442.1	0.91	0.19	26.5	26.6	12.6
2443.5	0.91	0.17	23.7	27.5	12.7
2444.9	0.97	0.13	20.1	26.3	12.6
2446.3	0.97	0.07	23.3	25.1	12.1
2447.7	0.93	0.09	21.7	24.8	11.8
2449.2	0.92	0.08	24.4	24.6	11.5
2450.7	0.86	0.03	27.3	23.5	11.1
2452.3	0.91	0.17	29.6	23.7	11.0
2453.9	0.82	0.09	31.4	21.5	10.7
2455.5	0.78	0.10	37.1	22.7	10.4
2457.2	0.56	0.07	36.8	20.7	8.3
2458.9	0.46	0.05	44.7	22.2	6.7
2460.7	0.40	0.03	45.4	21.0	5.8
2462.4	0.35	0.02	46.3	19.2	5.3
2464.1	0.28	0.03	51.0	21.3	4.7
2465.9	0.22	0.06	72.4	22.0	4.5
2467.6	0.33	0.03	82.7	19.8	5.4
2469.3	0.34	0.04	98.7	19.9	6.2
2471.1	0.39	0.06	96.1	22.1	7.1
2472.8	0.42	0.12	87.8	22.2	8.9
2474.4	0.69	0.25	87.6	20.7	11.5
2476.2	0.64	0.23	65.0	21.5	11.2
2477.9	0.71	0.17	44.1	21.2	10.8

2479.7	0.52	0.13	47.2	18.9	10.8
2481.5	0.77	0.25	25.9	21.4	10.7
2483.3	0.76	0.21	33.2	19.6	10.7
2485.0	0.83	0.20	33.4	19.3	10.9
2486.8	0.60	0.17	39.1	19.9	9.7
2488.6	0.62	0.12	60.6	24.3	8.9
2490.5	0.44	0.09	39.0	19.1	8.0
2492.4	0.46	0.06	42.2	17.7	7.2
2494.4	0.41	0.06	43.6	17.4	6.2
2496.4	0.41	0.09	47.7	18.4	6.7
2498.3	0.48	0.08	42.9	17.3	6.6
2500.2	0.52	0.12	46.4	20.8	7.2
2502.1	0.63	0.11	43.8	20.2	8.1
2503.9	0.59	0.17	69.2	21.4	9.7
2505.7	0.81	0.15	32.4	27.7	11.0
2507.6	0.68	0.16	41.0	27.1	11.8
2509.5	0.84	0.15	41.9	22.0	11.9
2511.5	0.68	0.02	37.6	19.7	11.0
2513.7	0.63	0.13	38.2	21.2	10.5
2515.9	0.58	0.06	40.2	20.9	10.0
2518.0	0.70	0.06	46.8	20.1	10.6
2520.2	0.67	0.05	52.7	18.5	10.9
2522.3	0.60	0.08	62.3	21.6	11.1
2524.4	0.75	0.08	70.4	22.9	11.5
2526.4	0.75	0.10	46.5	21.8	11.2
2528.6	0.70	0.10	63.5	21.4	11.2
2530.9	0.45	0.06	92.9	20.6	8.8
2533.5	0.44	0.07	108.3	18.5	7.7
2536.6	0.36	0.03	145.4	19.5	6.5
2539.9	0.30	0.05	198.9	16.3	5.7
2543.6	0.27	0.03	207.7	14.6	5.1
2547.5	0.24	0.03	210.8	16.2	4.8
2551.6	0.25	0.03	214.6	15.7	4.7
2555.6	0.22	0.06	211.3	12.7	4.7
2560.0	0.21	0.03	181.3	13.8	4.4

SI References

1. T. S. Liu, *Loess and the Environment*. (China Ocean Press, Beijing, 1985), pp. 1-251.
2. Z. An, The history and variability of the East Asian paleomonsoon climate. *Quaternary Sci. Rev.* **19**, 171-187 (2000).
3. X. Y. Zhang *et al.*, Sources of Asian dust and role of climate change versus desertification in Asian dust emission. *Geophys. Res. Lett.* **30**, 2272 (2003).
4. J. M. Sun, M. Y. Zhang, T. S. Liu, Spatial and temporal characteristics of dust storms in China and its surrounding regions, 1960-1999: Relations to source area and climate. *J. Geophys. Res-Atmos.* **106**, 10325-10333 (2001).
5. G. Kukla, Loess Stratigraphy in Central China. *Quaternary Sci. Rev.* **6**, 191-219 (1987).
6. B. A. Maher, Palaeoclimatic records of the loess/palaeosol sequences of the Chinese Loess Plateau. *Quaternary Sci. Rev.* **154**, 23-84 (2016).
7. S. C. Porter, Chinese loess record of monsoon climate during the last glacial-interglacial cycle. *Earth-Sci. Rev.* **54**, 115-128 (2001).
8. Z. An *et al.*, in *Late Cenozoic climate change in Asia,* Z. An, Ed. (Springer, London, 2014), pp. 23-143.
9. Z. H. An, G. J. Kukla, S. C. Porter, J. L. Xiao, Magnetic-susceptibility evidence of monsoon variation on the Loess Plateau of central China during the last 130,000 years. *Quaternary Res.* **36**, 29-36 (1991).
10. Z. Ding, Z. Yu, N. W. Rutter, T. Liu, Towards an orbital time-scale for Chinese loess deposits. *Quaternary Sci. Rev.* **13**, 39-70 (1994).
11. Z. T. Guo *et al.*, Summer monsoon variations over the last 1.2 Ma from the weathering of loess-soil sequences in China. *Geophys. Res. Lett.* **27**, 1751-1754 (2000).
12. S. C. Porter, Z. S. An, Correlation between climate events in the North-Atlantic and China during last glaciation. *Nature* **375**, 305-308 (1995).
13. J. C. Chow, J. G. Watson, D. Crow, D. H. Lowenthal, T. Merrifield, Comparison of IMPROVE and NIOSH carbon measurements. *Aerosol Sci. Tech.* **34**, 23-34 (2001).
14. Y. N. Han *et al.*, Evaluation of the thermal/optical reflectance method for quantification of elemental carbon in sediments. *Chemosphere* **69**, 526-533 (2007).
15. Y. M. Han, J. Marlon, J. J. Cao, Z. D. Jin, Z. S. An, Holocene linkages between char, soot, biomass burning and climate from Lake Daihai, China. *Global Biogeochem. Cycles* **26**, GB4017 (2012).
16. Y. M. Han *et al.*, The effect of acidification on the determination of elemental carbon, char-, and soot-elemental carbon in soils and sediments. *Chemosphere* **75**, 92-99 (2009).
17. J. C. Chow *et al.*, The DRI thermal/optical reflectance carbon analysis system: description, evaluation and applications in U.S. Air quality studies. *Atmos. Environ. Part A. General Topics* **27**, 1185-1201 (1993).
18. Y. M. Han *et al.*, Evaluation of the thermal/optical reflectance method for discrimination between char- and soot-EC. *Chemosphere* **69**, 569-574 (2007).

19. DRI, "DRI Standard Operating Procedure: DRI Model 2001 Thermal/Optical Carbon Analysis (TOR/TOT) of Aerosol Filter Samples – Method IMPROVE_A," (Desert Research Institute, Reno, NV 89506, 2005).
20. R. J. Yokelson, R. Susott, D. E. Ward, J. Reardon, D. W. T. Griffith, Emissions from smoldering combustion of biomass measured by open-path Fourier transform infrared spectroscopy. *J. Geophys. Res-Atmos.* **102**, 18865-18877 (1997).
21. T. T. van Leeuwen, G. R. van der Werf, Spatial and temporal variability in the ratio of trace gases emitted from biomass burning. *Atmos. Chem. Phys.* **11**, 3611-3629 (2011).
22. T. J. Christian *et al.*, Comprehensive laboratory measurements of biomass-burning emissions: 1. Emissions from Indonesian, African, and other fuels. *J. Geophys. Res-Atmos.* **108**, 4719 (2003).
23. D. E. Ward *et al.*, Smoke and fire characteristics for cerrado and deforestation burns in Brazil - Base-B experiment *J. Geophys. Res.* **97**, 14601-14619 (1992).
24. D. E. Ward *et al.*, Effect of fuel composition on combustion efficiency and emission factors for African savanna ecosystems. *J. Geophys. Res-Atmos.* **101**, 23569-23576 (1996).
25. R. J. Yokelson *et al.*, Coupling field and laboratory measurements to estimate the emission factors of identified and unidentified trace gases for prescribed fires. *Atmos. Chem. Phys.* **13**, 89-116 (2013).
26. A. A. May *et al.*, Aerosol emissions from prescribed fires in the United States: A synthesis of laboratory and aircraft measurements. *J. Geophys. Res-Atoms.* **119**, 11826-11849 (2014).
27. G. R. McMeeking *et al.*, Emissions of trace gases and aerosols during the open combustion of biomass in the laboratory. *J. Geophys. Res.* **114**, (2009).
28. C. A. Masiello, New directions in black carbon organic geochemistry. *Mar. Chem.* **92**, 201-213 (2004).
29. M. W. I. Schmidt, A. G. Noack, Black carbon in soils and sediments: Analysis, distribution, implications, and current challenges. *Global Biogeochem. Cycles* **14**, 777-793 (2000).
30. W. Permchart, V. I. Kouprianov, Emission performance and combustion efficiency of a conical fluidized-bed combustor firing various biomass fuels. *Bioresource Technol.* **92**, 83-91 (2004).
31. R. J. Yokelson *et al.*, Trace gas and particle emissions from open biomass burning in Mexico. *Atmos. Chem. Phys.* **11**, 6787-6808 (2011).
32. G. R. McMeeking *et al.*, Emissions of trace gases and aerosols during the open combustion of biomass in the laboratory. *J. Geophys. Res-Atoms.* **114**, D19210 (2009).
33. N. W. Brewer *et al.*, Fuel moisture influences on fire-altered carbon in masticated fuels: An experimental study. *J. Geophys. Res-Biogeo.* **118**, 30-40 (2013).
34. Y. M. Han *et al.*, Climate and fuel controls on North American paleofires: Smoldering to flaming in the late-glacial-Holocene transition. *Sci. Rep.* **6**, 20719 (2016).

35. E. A. Hoffa, D. E. Ward, W. M. Hao, R. A. Susott, R. H. Wakimoto, Seasonality of carbon emissions from biomass burning in a Zambian savanna. *J. Geophys. Res-Atmos.* **104**, 13841-13853 (1999).
36. Y. M. Han, S. C. Lee, J. J. Cao, K. F. Ho, Z. S. An, Spatial distribution and seasonal variation of char-EC and soot-EC in the atmosphere over China. *Atmos. Environ.* **43**, 6066-6073 (2009).
37. Y. M. Han, J. J. Cao, S. C. Lee, K. F. Ho, Z. S. An, Different characteristics of char and soot in the atmosphere and their ratio as an indicator for source identification in Xi'an, China. *Atmos. Chem. Phys.* **10**, 595-607 (2010).
38. E. D. Goldberg, *Black Carbon in the Environment*. (John Wiley & Sons, Inc., New York, 1985), pp. 1-198.
39. C.-H. Jeong *et al.*, Identification of the sources and geographic origins of black carbon using factor analysis at paired rural and urban sites. *Environ. Sci. Tech.* **47**, 8462-8470 (2013).
40. Y. Sun, S. C. Clemens, Z. An, Z. Yu, Astronomical timescale and palaeoclimatic implication of stacked 3.6-Myr monsoon records from the Chinese Loess Plateau. *Quaternary Sci. Rev.* **25**, 33-48 (2006).
41. Z. L. Ding *et al.*, Coeval changes in grain size and sedimentation rate of eolian loess, the Chinese Loess Plateau. *Geophys. Res. Lett.* **28**, 2097-2100 (2001).
42. L. E. Lisiecki, M. E. Raymo, A Pliocene-Pleistocene stack of 57 globally distributed benthic $\delta^{18}O$ records. *Paleoceanography* **20**, PA2007 (2005).
43. A. de Vernal, C. Hillaire-Marcel, Natural variability of Greenland climate, vegetation, and ice volume during the past million years. *Science* **320**, 1622-1625 (2008).
44. D. Oppo *et al.*, A $\delta^{13}C$ record of Upper North Atlantic Deep Water during the past 2.6 million years. *Paleoceanography* **10**, 373-394 (1995).
45. D. A. Hodell, K. A. Venz, C. D. Charles, U. S. Ninnemann, Pleistocene vertical carbon isotope and carbonate gradients in the South Atlantic sector of the Southern Ocean. *Geochem. Geophy. Geosy.* **4**, (2003).
46. D. Luthi *et al.*, High-resolution carbon dioxide concentration record 650,000-800,000 years before present. *Nature* **453**, 379-382 (2008).
